# Contractile forces at tricellular contacts modulate epithelial organization and monolayer integrity

**DOI:** 10.1038/ncomms13998

**Published:** 2017-01-13

**Authors:** Julie Salomon, Cécile Gaston, Jérémy Magescas, Boris Duvauchelle, Danielle Canioni, Lucie Sengmanivong, Adeline Mayeux, Grégoire Michaux, Florence Campeotto, Julie Lemale, Jérôme Viala, Françoise Poirier, Nicolas Minc, Jacques Schmitz, Nicole Brousse, Benoit Ladoux, Olivier Goulet, Delphine Delacour

**Affiliations:** 1 Cell Adhesion and Mechanics, Institut Jacques Monod, CNRS UMR7592, Paris Diderot University, 75205 Paris, France; 2 Department of Paediatric Gastroenterology, Hôpital Necker-Enfants Malades, Sorbonne Paris Cité, 75015 Paris, France; 3 Morphogenesis, Homoeostasis and Pathologies, Institut Jacques Monod, CNRS UMR7592, Paris Diderot University, 75013 Paris, France; 4 Department of Paediatric Anatomo-Pathology, Hôpital Necker-Enfants Malades, Sorbonne Paris Cité, 75015 Paris, France; 5 Membrane Dynamics and Mechanics of Intracellular Signaling Laboratory, Institut Curie–Centre de Recherche, PSL Research University, 75005 Paris, France; 6 Institut de Génétique et Développement de Rennes, CNRS UMR6290, 35000 Rennes, France; 7 Laboratoire de Microbiologie EA 4065, Faculté de Pharmacie, Université Paris Descartes, 75005 Paris, France; 8 Department of Pediatric Nutrition and Gastroenterology, Armand-Trousseau Hospital, Assistance Publique-Hôpitaux de Paris, Institute of Cardiometabolism and Nutrition, Pierre et Marie Curie University, 75012 Paris, France; 9 Department of Pediatric Gastroenterology, Assistance Publique-Hôpitaux de Paris, Robert Debré Hospital, Université Paris Diderot, Sorbonne Paris Cité, UMR843, 75019 Paris, France; 10 Cellular Spatial Organization, Institut Jacques Monod, CNRS UMR7592, Paris Diderot University, 75205 Paris, France; 11 Mechanobiology Institute, National University of Singapore, Singapore 117411, Singapore

## Abstract

Monolayered epithelia are composed of tight cell assemblies that ensure polarized exchanges. EpCAM, an unconventional epithelial-specific cell adhesion molecule, is assumed to modulate epithelial morphogenesis in animal models, but little is known regarding its cellular functions. Inspired by the characterization of cellular defects in a rare EpCAM-related human intestinal disease, we find that the absence of EpCAM in enterocytes results in an aberrant apical domain. In the course of this pathological state, apical translocation towards tricellular contacts (TCs) occurs with striking tight junction belt displacement. These unusual cell organization and intestinal tissue defects are driven by the loss of actomyosin network homoeostasis and contractile activity clustering at TCs, yet is reversed by myosin-II inhibitor treatment. This study reveals that adequate distribution of cortical tension is crucial for individual cell organization, but also for epithelial monolayer maintenance. Our data suggest that EpCAM modulation protects against epithelial dysplasia and stabilizes human tissue architecture.

## Abstract (web summary)

EpCAM is an unconventional epithelia-specific cell–cell adhesion molecule, that is mutated in the majority of cases of Congenital Tufting Enteropathy. Here the authors show that loss of EpCAM causes a concentration of contractile activity at tricellular junctions, leading to aberrant apical domain and tight junction displacement.

Constitutional dysmorphology of enterocytes leads to rare human congenital enteropathies. The MicroVillous Inclusion Disease (MVID) and the less studied Congenital Tufting Enteropathy (CTE) have both the common characteristic of being responsible for chronic diarrhoea, persistent during digestive rest and exacerbated by food uptake. MVID and CTE diseases are distinct from inflammatory bowel diseases, such as Crohn disease or autoimmune enteropathy that results from immune dysregulation^1^,^2^,^3^.

The CTE (MIM #613217), alternatively named intestinal epithelial dysplasia, leads to intestinal insufficiency soon after birth. No curative treatment is available, and the pathology is rapidly lethal unless palliative care, namely daily parenteral nutrition (that is, intravenous feeding, bypassing eating and digestion processes)^1^,^2^. CTE has an incidence estimated to 1/50,000–100,000 in Western Europe^4^. CTE intestinal epithelium displays unique morphological abnormalities, materialized by formation of aberrant focal stacks of pseudo-multilayered enterocytes on the villus, named ‘tufts'^5^ (Fig. 1a,b). At late stages, tufts can affect up to 70% of the villi^1^,^5^. CTE disease has been associated with pathogenic loss of function mutations of the *EPCAM* gene in 73% of the patients^3^,^6^,^7^.

EpCAM (Epithelial-Cell Adhesion Molecule) is a transmembrane glycoprotein that is expressed in various epithelia. Often used as an epithelial cancer marker in clinical studies, it has been primarily described as an unconventional Ca^2+^-independent homophilic CAM protein^8^,^9^, but clear molecular mechanisms for how EpCAM may regulate epithelium architecture are still lacking. Diverse models of EpCAM signalling functions have been proposed. The best characterized function of EpCAM concerns cell proliferation. Gires and colleagues^10^ showed that proteolytic cleaved intracellular fragment of EpCAM and its nuclear translocation is capable of directly modulating transcription factors. Moreover, EpCAM deprivation or overexpression has been proposed to influence bulk actin organization in epithelial thymic cells^11^. However, precise mechanisms mediating this effect remains to be found. EpCAM has also been reported to exhibit α-actinin-binding sites^9^, but these observations have not been pursued. In addition, whether EpCAM belongs to a well-described adhesion complex or constitutes an independent adhesion complex is unknown. A functional «connection» between EpCAM and E-cadherin has been proposed, with no direct physical interaction^12^,^13^. Several studies suggested a potential interplay between EpCAM and E-cadherin-based cell contact sites. Overexpression of EpCAM interferes with E-cadherin-based cell adhesion, and EpCAM has been considered as an antagonist of intercellular adhesion^12^. Knockdown of EpCAM in Zebrafish and Xenopus epidermis caused perturbations of E-cadherin stabilization at adherens junctions (AJs)^14^,^15^. Recently, EpCAM has been reported to be dispensable for direct cell–cell adhesion or cell-substrate adhesion *per se*^16^. Thus EpCAM functions are probably very different from what was assumed, and, importantly, no mechanism for how EpCAM deficiency may lead to enterocyte dysfunction has been proposed to date.

Here we characterize cellular defects related to EpCAM loss in CTE intestinal biopsies and in EpCAM-depleted Caco2 cells. We find that the absence of EpCAM causes unusual epithelial individual cell organization defects, together with perturbation of the monolayered intestinal epithelium. We show that these abnormalities stem from an inappropriate actomyosin activity at tricellular contacts (TCs), thus providing a new function for EpCAM in contractile force patterning in epithelium.

## Results

### EpCAM is required for intestinal brush border integrity

**Figure 1:**
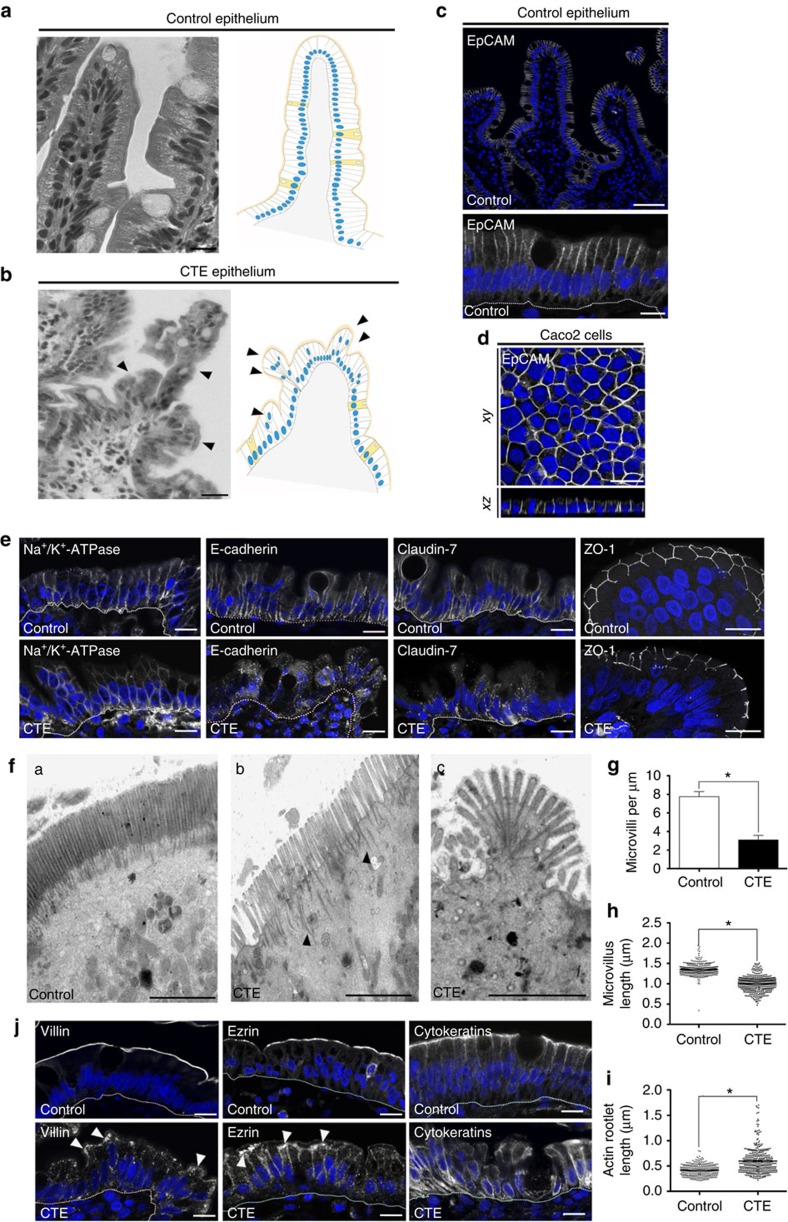
Cell organization defects occur in the intestinal epithelium of CTE patients. (**a**,**b**) Histological analysis of hematoxylin–eosin stained paraffin sections of duodenal biopsies from control (**a**) and CTE patients (**b**). Scale bars, 10 μm. For each condition, a corresponding scheme of the epithelial organization is presented. CTE intestinal epithelium displays tufts (black arrowheads). (**c**,**d**) Confocal microscopy analysis of EpCAM distribution in duodenal control biopsy (**c**) or in Caco2 cells (**d**). Intestinal epithelium baseline is demarcated by dotted white line (**c**). Scale bars, upper panel 50 μm (**c**), lower panel 10 μm (**c**) and 10 μm (**d**). (**e**), Confocal microscopy analyses of Na^+^/K^+^-ATPase, E-cadherin, claudin-7 and ZO-1 distribution in control or CTE biopsies. *N*_(Control)_=6 biopsies, *N*_(CTE)_=6 biopsies. Scale bars, 10 μm. (**f**) Transmission electron microscopy (TEM) ultrastructural analysis of enterocyte apical membranes in control (**a**) and CTE (**b**,**c**) biopsies, showing actin rootlets (*black arrowheads*). Scale bars, 5 μm. *N*_(Control)_=3 biopsies, *N*_(CTE)_=3 biopsies. (**g**) Statistical analysis of microvillus density in control and CTE enterocytes. *t* test, *P<0.0004. (**h**) Statistical analysis of microvillus length in control and CTE enterocytes. Control(MV length)=1.35±0.007 μm, CTE(MV length)=1.001±0.009 μm. *t*-test, *P-value<0.0001. *N*_(Control)_=3 biopsies, *n*_(Control)_=352 microvilli, *N*_(CTE)_=3 biopsies, *n*_(CTE)_=400 microvilli. (**i**) Statistical analysis of the actin rootlet length in control and CTE enterocytes. Control(length)=0.416±0.006 μm, CTE(length)=0.596±0.01 μm. *N*_(Control)_=3 biopsies, *n*_(Control)_=309 actin rootlets, *N*_(CTE)_=3 biopsies, *n*_(CTE)_=394 actin rootlets. *t*-test, *P<0.0001. Values are mean±s.e.m. (**j**) Confocal microscopy analyses of villin, ezrin and cytokeratins distribution in control or CTE biopsy sections. *N*_(Control)_=12 biopsies, *N*_(CTE)_=6 biopsies. Scale bars, 10 μm. Nuclei were detected by Hoechst 33342 staining (blue).

To understand EpCAM role(s), we first analysed *EPCAM* mutated CTE enterocytes. Since EpCAM is distributed at lateral membranes in human enterocytes (Fig. 1c,d), we first focused on the distribution of cell–cell adhesion complexes. While no difference was observed for the Na^+^/K^+^-ATPase ionic pump (Fig. 1e), E-cadherin were barely detected at lateral membranes, but instead appeared at numerous cytoplasmic-positive compartments in *EPCAM*-mutated patients (Fig. 1e). Weakened cell–cell contacts and numerous cellular interdigitations at lateral membranes were also noticed (Supplementary Fig. 1A), indicating a loss of stability of E-cadherin-based cell adhesion complexes at CTE lateral membranes, as previously reported in the Zebrafish skin^14^, in Xenopus^15^ and in EpCAM mutant mice^17^. In addition, lateral membrane distribution of claudin-7, a component of the paracellular barrier permeability^18^, was impaired in CTE enterocytes (Fig. 1e), as seen in EpCAM mutant mice^19^. This suggests defects in barrier permeability in CTE epithelium, as previously described in EpCAM-silenced cells or EpCAM mutant mice^19^,^20^. Moreover, distribution of the tight junction associated protein ZO-1 revealed that tight junction belts were present, but displayed a distorted shape in CTE cells (Fig. 1e). At apical membranes, CTE brush border exhibited microvillus rarefaction (Fig. 1f,g), variable microvillous atrophy (Fig. 1f,h), as well as length increase and contraction of actin rootlets (Figs 1f(b,c) and 1i and Fig. 1f(c), respectively), resulting in a fan-like shaped brush border. Brush border structural components, such as villin and ezrin, partially disappeared from the brush border and accumulated in the terminal web area (Fig. 1j, white arrowheads). Terminal web cytoskeletal elements, such as cytokeratins, were severely displaced from the apical cortex (Fig. 1j). In addition, abnormal presence of numerous intracellular organelles was noted in the terminal web area (Supplementary Fig. 1Ba,b, white arrowheads), as well as apical plasma membrane herniations (Supplementary Fig. 1Bc, black stars). Altogether, these data show that the brush border is destructured on EpCAM loss of function in CTE intestines. As a result, the brush border-associated lactase phlorizin hydrolase became accumulated intracellularly in CTE biopsies compared with control samples (Supplementary Fig. 1C). These results show that EpCAM loss of function impairs the integrity of the entire brush border organelle and consequently the functional enterocyte terminal differentiation in human intestine. Although tight junction distribution was in favour of a conservation of the apico-basal barrier *per se* in human CTE biopsies, brush border components were massively relocated at lateral membranes in CTE biopsies (Fig. 1j), suggesting that epithelial organization was affected in an unusual manner. These data suggest that EpCAM plays a major role in maintaining epithelial integrity.

### EpCAM silencing causes apical domain expansion at TCs

**Figure 2:**
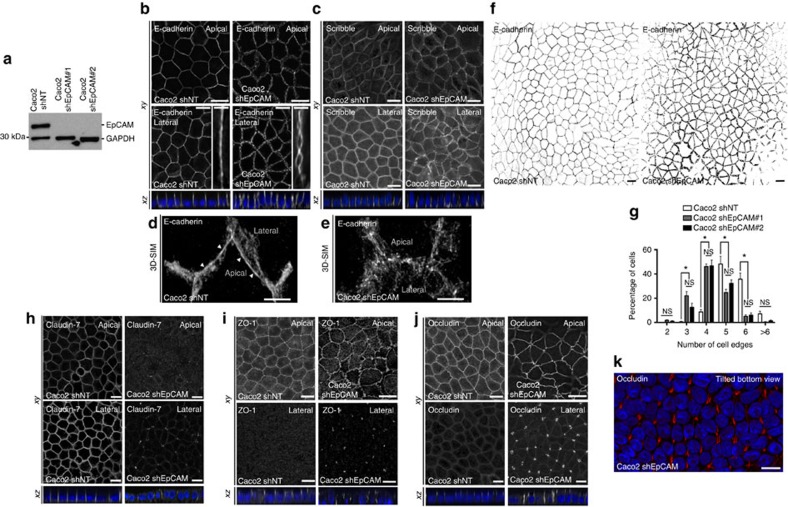
EpCAM silencing leads to unusual tight junction positioning defects at tricellular junctions. (**a**) Western blot analysis of the EpCAM expression level in control (*Caco2 shNT*) or EpCAM-deprived (*Caco2 shEpCAM #1* and *Caco2 shEpCAM #2*) clones. GAPDH was used as loading control. (**b**,**c**) Confocal microscopy analysis of E-cadherin (**b**) and Scribble (**c**) on the apical or lateral sides in control and EpCAM-depleted cells. Scale bars, 5 μm. Nuclei were detected with Hoechst 33342 staining (blue). (**d**,**e**) 3D-SIM microscopy analysis of E-cadherin localization in control (**d**) and EpCAM-deprived (**e**) cells. Scale bars, 2 μm. (**f**) Cell shape analysis after E-cadherin immunostaining in control or EpCAM-deprived cells. Scale bars, 5 μm. (**g**), Statistical analysis of polygonal shape in control or EpCAM-deprived cells. Three independent experiments were carried out. *N* (Caco2shNT)=517 cells, *N* (Caco2shEpCAM#1)=550 cells, *N* (Caco2shEpCAM#2)=427 cells. Caco2 shNT cells with two cell edges=0%, three cell edges=0.455±0.787%, four cell edges=8.673±3.406%, five cell edges =48.154±11.123%, six cell edges=35.652±8.888%, more than six cell edges=7.067±3.851 cells%. Caco2 shEpCAM#1 cells with two cell edges=1.822±0.733 cells%, three cell edges=22.031±5.906%, four cell edges=46.061±3.851%, five cell edges=24.728±4.344%, six cell edges=5.027±2.210%, more than six cell edges=0.330±0.572%. Caco2 shEpCAM#2 cells with two cell edges=0.966±0.848%, three cell edges=12.542±5.942%, four cell edges=46.739±8.082%, five cell edges=32.319±4.777%, six cell edges=6.098±3.412%, more than six cell edges=1.336±1.261%. Unpaired *t*-tests, *P<0,0001. Values are mean±s.e.m. (**h**–**j**) Confocal microscopy analysis of claudin-7, ZO-1 and occludin on the apical or lateral sides in control and EpCAM-depleted cells. (**k**) Confocal analysis of occludin (red) location at TCs in EpCAM-deprived cells, 3D rendering of *z*-stacks. Tilted bottom view is presented. Scale bars, 5 μm. Nuclei were detected with Hoechst 33342 staining (blue).

To further study EpCAM cellular function(s), we generated stable human Caco2 clones silenced for EpCAM (Fig. 2a; Supplementary Fig. 2A–B). We first analysed cell–cell adhesion complexes. E-cadherin ladder-like patterns were noticed at bicellular lateral membranes (Fig. 2b), and apical AJ belt appeared punctuated in EpCAM-deprived cells (Fig. 2d,e, white arrowheads). EpCAM loss led to the presence of cell adhesion fractures at lateral membranes. To test specifity of these abnormalities, we performed rescue experiments by transfecting an EpCAM-GFP short hairpin RNA (shRNA)-resistant construct in EpCAM-depleted cells. Green fluorescent protein (GFP) construct has been used in parallel as a control (Supplementary Fig. 3A,B). EpCAM re-expression in EpCAM-silenced cells caused the disappearance of cell adhesion fractures (Supplementary Fig. 3A,B). Moreover, E-cadherin staining revealed that honeycomb-like cell shape was lost in absence of EpCAM in epithelial cells (Fig. 2f,g), and strongly suggested that an epithelial disequilibrium occurs within EpCAM mutated monolayers^21^,^22^. In addition to E-cadherin-based defects, the expression level of claudin-7 strongly decreased (Supplementary Fig. 2A,D), and its subcellular distribution was significantly modified on EpCAM loss (Fig. 2h). These results show that cell–cell adhesion is compromised in EpCAM-mutated cells. Nevertheless, basal polarity was not perturbed since Scribble, a component of the basolateral protein complex Scribble/Lethal Giant larvae/Disc large^23^, remained laterally located in the absence of EpCAM (Fig. 2c). In conclusion, in spite of the presence of discrete adhesion fractures and cell shape changes, bonafide basolateral determination was not grossly affected.

Moreover, we tested tight junction integrity in absence of EpCAM. Tight junction belts were present at apical-sides, but appeared deformed at bicellular contacts on EpCAM silencing (Fig. 2i,j), as observed in CTE biopsies (Fig. 1e). An increase of occludin expression occurred in EpCAM-deprived cells (Supplementary Fig. 2A,C), and an intriguing enrichment of tight junction components took place at TCs (Fig. 2i,j). The tight junction belt was distorted toward basal-side at TCs, creating a regular tube (Fig. 2k). EpCAM rescue caused the disappearance of tight junction belt distortion at TCs, and restored cortical tight junction belt positioning (Supplementary Fig. 3C,D). These results show that tight junction belt positioning was severely compromised on EpCAM loss.

**Figure 3:**
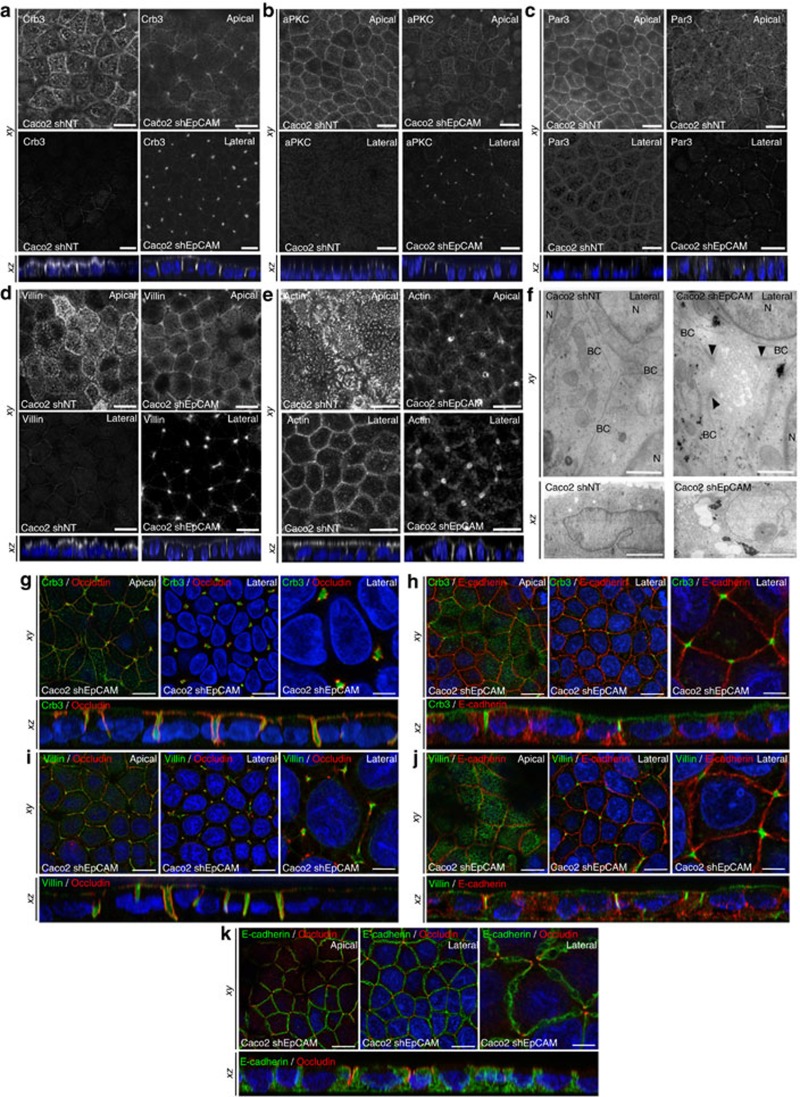
Apical domain is unusually displaced at tricellular junctions through EpCAM silencing. (**a**–**e**) Confocal microscopy analysis of Crb3 (**a**), aPKC (**b**), Par3 (**c**), villin (**d**) and actin (**e**) on the apical or lateral sides in control (*Caco2 shNT*, left panels) and EpCAM-depleted (*Caco2 shEpCAM*, right panels) cells. *xy* and *xz* views are presented showing the relocated apical markers at tricellular junctions. Scale bars, 5 μm. (**f**) TEM ultrastructural analysis of tricellular contacts in control (*Caco2 shNT*, left) and EpCAM-depleted (*Caco2 shEpCAM*, right) cells, showing microvilli at tricellular contacts. Arrowheads point to TJs. Longitudinal (*xy*) and transversal views (*xz*) are presented. BC, bicellular contact, N, nucleus. Scale bars *xy*, 1 μm and *xz*, 2 μm. (**g**–**k**) Confocal microscopy analysis of double immunostainings for Crb3 (green) and occluding (red) (**g**), Crb3 (green) and E-cadherin (red) (**h**), villin (green) and occluding (red) (**i**), villin (green) and E-cadherin (red) (**j**), and E-cadherin (green) and occluding (red) (**k**) on the apical or lateral sides in EpCAM-depleted (*Caco2 shEpCAM*) cells. *xy* and *xz* views are presented. Scale bars, 5 μm; high magnifications, 2 μm. Nuclei were detected with Hoechst 33342 staining (blue).

In parallel, we investigated apical polarity on EpCAM loss. Apical polarity players Crb3, aPKC and Par3 (ref. 23; Fig. 3a–c), and microvillous core components, such as villin and actin (Fig. 3d,e), were barely detectable at the apical domain of EpCAM-silenced cells, but instead massively relocated laterally at TCs. However, neither Par3 nor microvillous component ezrin expression was significantly perturbed on EpCAM loss (Supplementary Fig. 2A,E–F). Accordingly, with apical marker mislocalization, microvilli were formed along TC vertices (Fig. 3f). At higher resolution, tight junction and apical protein populations were not intermixed at TCs, where tight junction components rather embraced apical protein pools (Fig. 3g–k). Hence an apico-basal border protein complex did still exist in EpCAM-deprived enterocytes, but was aberrantly distorted towards atypical TC vertices with the consecutive appearance of extended apical domains (EADs). On EpCAM-GFP transfection in EpCAM-depleted cells, Par3 and villin were not anymore located at TCs and relocated at the apical membrane, showing that EADs faded away and cortical apical domain was recovered after EpCAM rescue (Supplementary Fig. 4). Altogether, these results suggest that EpCAM loss of function impairs tight junction complex and apical domain positioning, and that EpCAM is required for proper epithelial cell organization.

### Loss of tricellulin does not recapitulate EpCAM silencing

**Figure 4:**
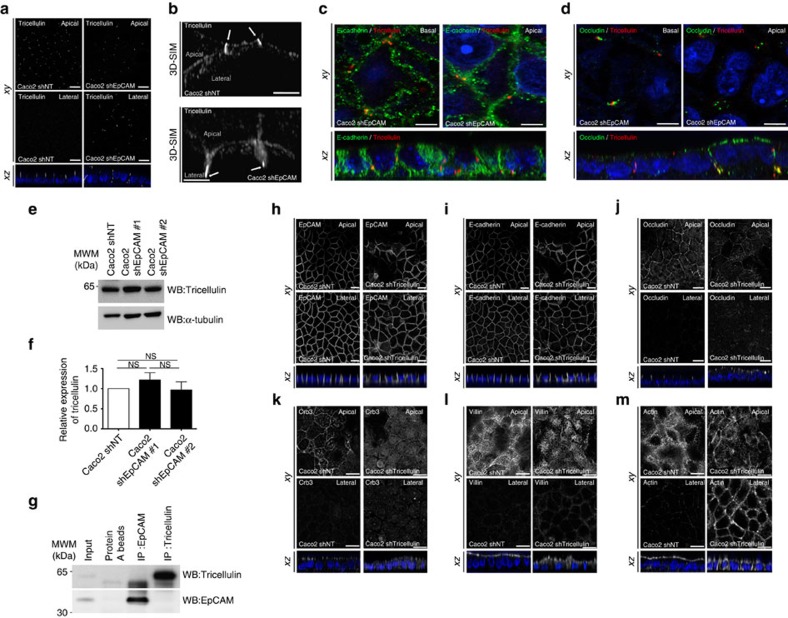
Tricellulin silencing does not lead to apical domain displacement at tricellular junctions. (**a**) Confocal microscopy analysis of tricellulin distribution on the apical (*Apical*, upper panels) or lateral (*Lateral*, lower panels) side in control (*Caco2 shNT*, left panels) and EpCAM-depleted (*Caco2 shEpCAM*, right panels) cells. *xy* and *xz* views are presented. Scale bars, 5 μm. (**b**) 3D-SIM microscopy analysis of tricellulin localization in control (*Caco2 shNT*) and EpCAM-deprived (C*aco2 shEpCAM*) cells. While tricellulin was focused at the most apical part of the tricellular junctions in control cells, it was instead concentrated at the bottom of aberrant tricellular junctional tubes in the absence of EpCAM (white arrows). Scale bars, 2 μm. (**c**,**d**) Confocal microscopy analysis of double immunostainings for E-cadherin (green) and tricellulin (red) (**c**), occludin (green) and tricellulin (red) (**d**), on the basal or apical sides in EpCAM-depleted (*Caco2 shEpCAM*) cells. *xy* and *xz* views are presented. Scale bars, 2 μm. (**e**) Western blot analysis of the level of tricellulin expression in control (*Caco2 shNT*) or EpCAM-deprived (*Caco2 shEpCAM #1* and *Caco2 shEpCAM #2*) clones. α-tubulin was used as loading control. (**f**) Statistical analysis of tricellulin amounts relative to α-tubulin amounts in control (*Caco2 shNT*) or EpCAM-deprived (*Caco2 shEpCAM #1* and *Caco2 shEpCAM #2*) clones. The analysis was performed based on three independent experiments. Percentage of tricellulin expression in control cells=100%, in Caco2 shEpCAM#1 cells=121.8±0.18%, and in Caco2 shEpCAM#2=96.8±0.20%. Three independent experiments were used, and statistical significance was determined one-way analysis of variance and Tukey's multiple comparison tests. (**g**) Western blot detection of tricellulin (upper panel) or EpCAM (lower panel) after immunoprecipitation of EpCAM or tricellulin from control Caco2 cell extracts. (**h**–**m**) Confocal microscopy analysis of EpCAM, E-cadherin, occluding, Crb3, villin and actin distribution on the apical (*Apical*, upper panels) or lateral (*Lateral*, lower panels) side in control (*Caco2 shNT*, left panels) and tricellulin-depleted (*Caco2 shTricellulin*, right panels) cells. *xy* and *xz* views are presented. Scale bars, 5 μm. Nuclei were detected with Hoechst 33342 staining (*blue*).

TCs have been recently described as narrow tubes at the contact of three neighbouring cells, and we wondered whether a perturbation of TCs might be responsible of EAD formation. We first tested the distribution of tricellulin, a major component of these junctions^24^,^25^. In EpCAM-silenced cells, tricellulin puncta were aberrantly displaced towards the bottom of tubes embraced by tight junction components at TCs (Fig. 4a–d), suggesting an impairement of TC positioning. This result prompted us to test a putative functional interaction between EpCAM and tricellulin. We first tested the expression level of tricellulin, and we did not observe a significant change in tricellulin amounts on EpCAM loss (Fig. 4e,f). We also analysed their interaction by immunoprecipitation followed by western blot (WB). We found that tricellulin does not co-immunoprecipitate with EpCAM, and vice versa (Fig. 4g), demonstrating that EpCAM and tricellulin may not physically interact. We next analysed cellular defects induced by tricellulin silencing (Supplementary Fig. 5A,B). While rosette formation occurred as previously reported^26^, EpCAM and E-cadherin localization were not grossly affected (Fig. 4h,i). The same observation was made for the tight junction component occludin and the apical polarity player Crb3, although a decrease of apical signal and a slight increase of cytosolic staining could be observed in tricellulin-silenced cells (Fig. 4j,k). Moreover, villin immunostaining was less regular at the apical domain and actin became less present in microvilli, but instead enriched at lateral borders (Fig. 4l,m). Tricellulin silencing also led to massive redirection of actin cytoskeleton along lateral membranes (Fig. 4m). Importantly, no EAD was formed in absence of tricellulin (Fig. 4j,m). No significant change in the expression of the hallmark proteins we analysed was noticed after tricellulin deprivation (Supplementary Fig. 5). These results demonstrate that tricellulin silencing in Caco2 cells does not significantly affect the global organization of individual cells, but causes a perturbation of the brush border structure and a relocation of the actin network along lateral membranes. We conclude that tricellulin depletion does not mimic EpCAM loss in Caco2 cells.

### Local hypercontractility occurs at TCs on EpCAM loss

**Figure 5:**
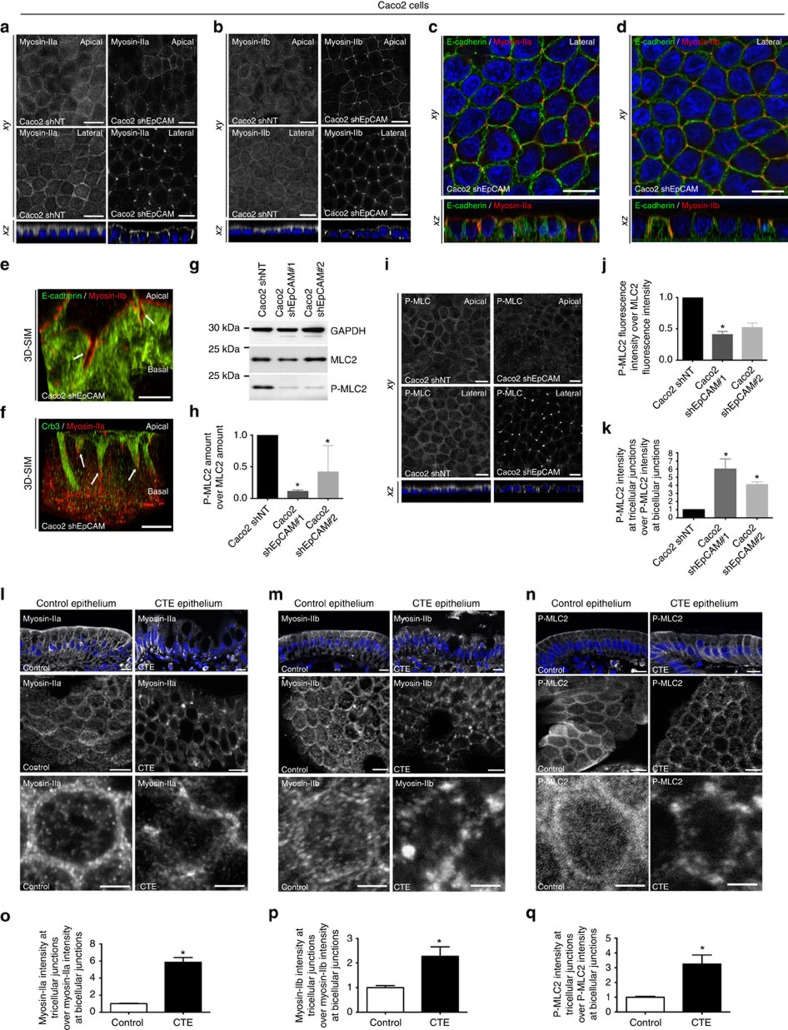
Exacerbation of contractile forces at TCs on EpCAM loss. (**a**,**b**) Distribution of myosin-IIa and -IIb in control and EpCAM-depleted cells. Scale bars, 5 μm. (**c**,**d**) Distribution of E-cadherin and myosin-IIa, or E-cadherin and myosin-IIb in EpCAM-depleted cells. Scale bars, 5 μm. (**e**,**f**) 3D-SIM analysis of E-cadherin and myosin-IIb, or Crb3 and myosin-IIa in EpCAM-deprived cells. Scale bars, 5 μm. (**g**) Western blot analysis of MLC2 and P-MLC2 amounts in control and EpCAM-depleted cells. GAPDH was used as loading control. (**h**) Quantification of P-MLC2 amount relative to MLC2 in control and EpCAM-depleted cells. One-way ANOVA with Dunnett's test, *P<0.01. Caco2shNT=1, Caco2shEpCAM#1=0.11±0.2, Caco2shEpCAM#2=0.42±0.2. (**i**) Distribution of P-MLC2 in control or EpCAM-silenced cells. Scale bars, 5 μm. (**j**) Quantification of total P-MLC2 immunofluorescence intensity relative to MLC2 immunofluorescence intensity in control or EpCAM-depleted cells. One-way analysis of variance test with Dunnett's test, *P=0.007, **P=0.002. *n*_(Caco2shNT)_=30 cells, *n*_(Caco2shEpCAM#1)_=30, *n*_(Caco2shEpCAM#2)_=30. Caco2shNT=1, Caco2shEpCAM#1=0.4±0.05, Caco2shEpCAM#2=0.52±0.07. (**k**) Quantification of P-MLC2 immunofluorescence intensity at TCs relative to immunofluorescence intensity at bicellular junctions (BJs) in control or EpCAM-depleted cells. One-way analysis of variance with unpaired *t*-test, *P=0.0001. *n*_(Caco2shNT)_=30 cells, *n*_(Caco2shEpCAM#1)_=28, *n*_(Caco2shEpCAM#2)_=30. Caco2shNT=1, Caco2shEpCAM#1=6.01±1.24, Caco2shEpCAM#2=4.1±0.33. (**l**–**n**) Distribution of myosin-IIa, -IIb and P-MLC2 in control or CTE biopsies. Scale bars, 5 μm. *N*_(Control)_=3 biopsies, *N*_(CTE)_=3. (**o**–**q**) Quantification of myosin-IIa, -IIb or P-MLC2 immunofluorescence intensity at TCs relative to immunofluorescence intensity at BJs in control or CTE biopsies. *N*_(Control)_=3 biopsies, *N*_(CTE)_=3. Unpaired *t*-test, (**o**) *P<0.0001, (**p**) *P=0.0062, (**q**) *P=0.0016. *n*_(Control myosin-IIa)_=37 cells, *n*_(CTE myosin-IIa)_=29, *n*_(Control myosin-IIb)_=14, *n*_(CTE myosin-IIb)_=14, *n*_(Control P-MLC2)_=15, *n*_(CTE P-MLC2)_=21. Myosin-IIa_(Control)_=1±0.04, myosin-IIa_(CTE)_=5.86±0.54, myosin-IIb_(Control)_=1±0.08, myosin-IIb_(CTE)_=2.27±0.39, P-MLC2_(Control)_=1±0.07, P-MLC2_(CTE)_=3.25±0.62. For quantification, three independent replicates have been performed. Values are mean±s.e.m. Nuclei were stained with Hoechst 33342.

How might EpCAM act at the molecular level? The large panel of observed defects (that is, brush border contraction, tight junction belt irregularity, TC displacement, cell shape defects) converged to a possible participation of EpCAM in the cortical contractility generated at cell junctions^27^,^28^,^29^. On the inner surface of lateral membranes, actin filaments are associated with E-cadherin-based cell adhesion complexes and generate most of the intracellular mechanical forces generated through their interaction with the motor proteins myosin-IIa and IIb (ref. 30). We tested if perturbation of the actomyosin activity could account for cellular defects observed in EpCAM-deprived cells. Strikingly, in EpCAM-silenced cells, we found that the homogeneous lateral distribution of myosins-IIa and -IIb was lost and that those motor isoforms were massively redirected to atypical TC vertices (Fig. 5a–d), where they covered the EADs (Fig. 5e,f). Proper cortical and lateral localization of myosin-IIa and -IIb was restored on EpCAM rescue (Supplementary Fig. 6A–D). The ability of the relocated actomyosin to generate cell contractility in mutant cells was further probed by analysing the phosphorylation state of the myosin regulatory light chain (P-MLC2)^31^. Using both biochemical and immunofluorescence approaches, we observed that the amount of P-MLC2 was markedly reduced in absence of EpCAM (Fig. 5g–j). The same tendency was observed using an antibody directed against double-phosphorylated MLC2 (Supplementary Fig. 7). However, a local concentration of the remaining pool of P-MLC2 was observed at TCs in mutant Caco2 cells (Fig. 5i,k). Re-expression of an EpCAM-GFP shRNA-resistant construct could restore amounts of P-MLC2 comparable to those measured in control cells (Supplementary Fig. 8A,B), and suppressed P-MLC2 accumulation at TCs (Supplementary Fig. 8C,D). Similar mislocalization and accumulation of myosin-II isoforms and P-MLC2 at TCs were observed in CTE biopsies (Fig. 5l–q). Thus, the absence of EpCAM may lead to a local hypercontractility at TCs in epithelial cells, suggesting that the proper patterning of the actomyosin network at cell–cell contacts depends on EpCAM.

### Actomyosin perturbation drives apical domain mispositioning

**Figure 6:**
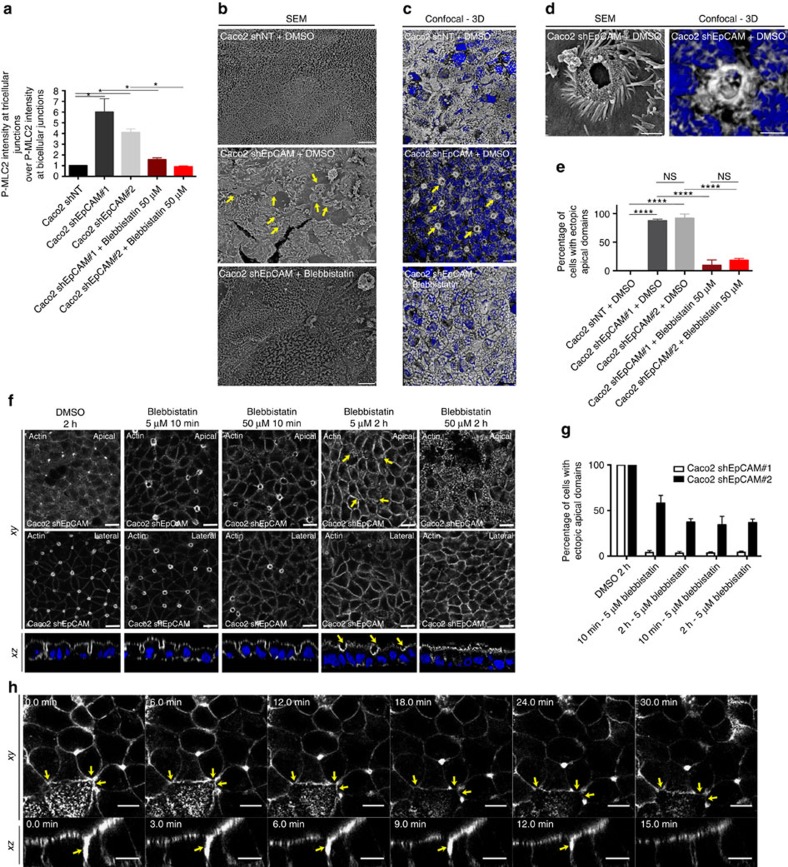
Apical domain positioning is restored on cell contractility decrease. (**a**) Quantification of P-MLC2 immunofluorescence intensity at TCs relative to immunofluorescence intensity at BJs in control, EpCAM-depleted or 2h-blebbistatin-treated EpCAM-depleted cells. One-way analysis of variance with unpaired *t*-test, *P<0.05. *n*_(Caco2shNT)_=30 cells, *n*_(Caco2shEpCAM#1)_=28, *n*_(Caco2shEpCAM#2)_=30, *n*_(Caco2shEpCAM#1+Blebbistatin 50 μM)_=31, *n*_(Caco2shEpCAM#2+Blebbistatin 50 μM)_=30. Caco2shNT=1, Caco2shEpCAM#1=6.01±1.24, Caco2shEpCAM#2=4.1±0.33, Caco2shEpCAM#1+Blebbistatin 50 μM=1.57±0.17, Caco2shEpCAM#2+Blebbistatin50 μM=0.91±0.06. (**b**) SEM analyses of apical surfaces of DMSO-treated control, EpCAM-depleted or EpCAM-depleted cells treated with blebbistatin for 2 h. Scale bars, 4 μm. (**c**) Confocal analysis and 3D rendering of *z*-stacks of actin location at TCs in DMSO-treated control, EpCAM-depleted or EpCAM-depleted cells treated with blebbistatin for 2 h. Scale bars, 4 μm. (**d**) High magnifications of EADs from SEM or confocal and 3D rendering analyses. Scale bars, 1 μm. (**e**) Quantification of EADs on DMSO or blebbistatin treatment in Caco2shNT or shEpCAM cells. One-way ANOVA with Tukey's test, ****P<0.0001. *n*_(Caco2shNT+DMSO)_=107 cells, *n*_(Cao2shEpCAM#1+DMSO)_=105, *n*_(Caco2shEpCAM#2+DMSO)_=91, *n*_(Caco2shEpCAM#1+Blebbistatin)_=96, *n*_(Caco2shEpCAM#2+Blebbistatin)_=81. EAD-positive Caco2shNT cells=0%, EAD-positive Caco2shEpCAM#1+DMSO cells=87.41±1.64%, EAD-positive Caco2shEpCAM#1+DMSO cells=91.98±4.21%, EAD-positive Caco2shEpCAM#1+blebbistatin cells=9.55±5.46%, EAD-positive Caco2shEpCAM#1+DMSO=18.43±1.83%. (**f**) Confocal analysis of actin distribution and brush border formation in Caco2shEpCAM cells. Cells were treated for 2 h with DMSO, or for 10 min or 2 h with 5 or 50 μM of blebbistatin. Scale bars, 5 μm. (**g**) Quantification of EADs on DMSO or blebbistatin treatment in Caco2 shEpCAM cells. *N*(2 h, DMSO)=5,467 cells, *N*(10 min, 5 μΜ)=3,598, *N*(10 min, 50 μΜ)=4,032, *N*(2 h, 5 μΜ)=289, *N*(2 h, 50 μΜ)=4,416. *t*-test, *P*<0.0001. (**h**) Analysis of lateral membrane behaviour in EpCAM-deprived Caco2 cells during 50 μM blebbistatin treatment and time-lapse acquisitions. Lateral membranes have been stained with SirActin labelling. Scale bars, 10 μm. For quantification, three independent replicates have been performed. Values are mean±s.e.m. Nuclei were stained with Hoechst 33342.

To determine whether perturbation of the cell contractility pattern could be responsible for cellular defects described above, we reduced myosin-II activity in EpCAM-silenced clones using blebbistatin^32^. Strikingly, blebbistatin-treated EpCAM-depleted cells recovered P-MLC2 location at bicellular junctions (Fig. 6a). We next tested the presence of EADs after blebbistatin treatment at the ultrastructural level using scanning electron microscopy (SEM; Fig. 6b,d,e). Brush borders regularly appeared at the apical surface of the monolayer in dimethylsulphoxide (DMSO)-treated control cells (Fig. 6b). The apical surface of DMSO-treated EpCAM-deprived cells displayed irregular or even non-existent brush border. Instead, we observed microvilli in vortex assemblies at TCs, likely EAD structures (Fig. 6b, yellow arrowheads). The particular arrangement of microvilli in EADs at TCs was further confirmed by actin staining of DMSO-treated mutant cells and three dimensional (3D) rendering of confocal *z*-stacks (Fig. 6c–d). Blebbistatin treatment caused the disappearance of EADs in EpCAM-silenced cells, and microvilli could be distinguished at the apical surface again (Fig. 6b,c,e). Furthermore, time course analysis showed that microvilli were progressively observed at cell corners under 5 and 50 μM blebbistatin treatment for 2 h (Fig. 6f,g, yellow arrowheads) and an impressive cortical restoration of the brush border was obtained on a 2 h treatment with 50 μM blebbistatin (Fig. 6f). In addition, time-lapse imaging of lateral membranes on blebbistatin treatment showed that bicellular junctions arose from EADs at TCs (Fig. 6h, yellow arrowheads). Moreover, *xz* views highlighted the progressive reduction of the EADs from the basal-side to the apical-side (Fig. 6h, yellow arrowheads). These data suggest that a myosin-II-dependent inward pulling force may be produced at TCs in EpCAM-depleted cells, from the apical-side to the basal-side, plausibly in a similar manner of what has been proposed for extruding apoptotic cells and epithelial tissue bending^33^. In addition, enrichment of actomyosin and apical markers was not observed anymore at TCs after blebbistatin treatment of EpCAM-silenced cells (Supplementary Fig. 9A–C). Brush border was partially restored at the cortical surface (Fig. 6f; Supplementary Fig. 9B). Occludin recovered a correct junctional distribution at the apical surface, in spite of a dotty pattern, possibly as a consequence of myosin inhibition (Supplementary Fig. 9D). Tricellulin was not detected anymore at tricellular lateral membranes (Supplementary Fig. 9E). Cell adhesion fractures were not observed, and honeycomb-like cell shape was progressively restored in blebbistatin-treated EpCAM-depleted cells (Supplementary Fig. 9F,G). These data show that the translocation of the apical compartment and EAD formation result from excessive contractile activity at TCs and consecutive shrinking of bicellular junctions on EpCAM loss.

### Actomyosin disorganization causes loss of monolayered state

**Figure 7:**
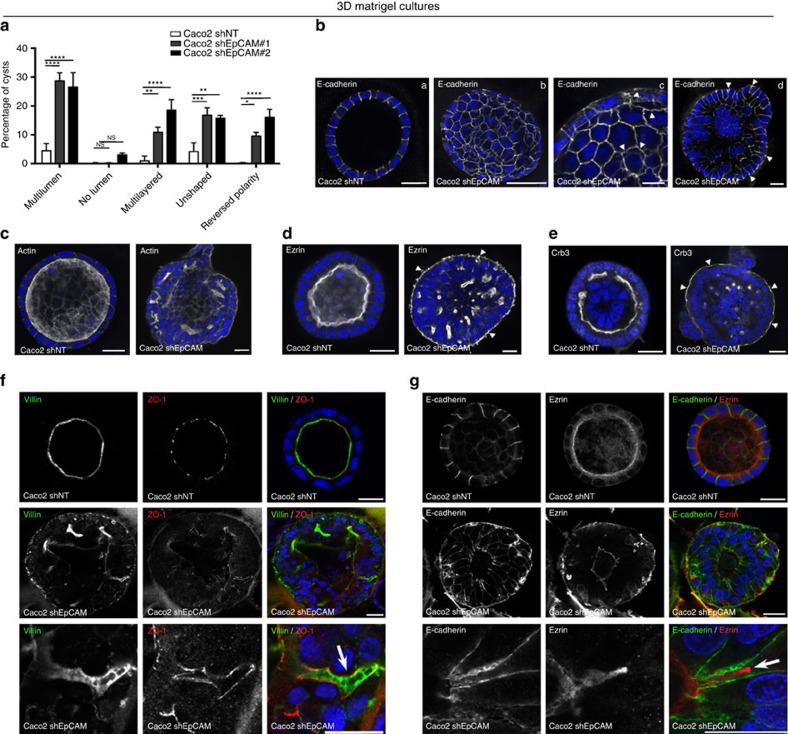
Epithelial organization in 3D Matrigel cultures is perturbed on EpCAM loss. (**a**) Statistical analysis of cyst morphology in Caco2 shNT (white), Caco2 shEpCAM#1 (grey) and Caco2 shEpCAM#2 (black) cells. Three independent experiments were performed. *N*_(Caco2shNT)_=779 cysts, *N*_(Caco2shEpCAM#1)_=1,049 cysts, *N*_(Caco2shEpCAM#2)_=1,686 cysts. Unpaired *t*-tests, ****P<0.0001, ***P<0.001, **P<0.01, *P<0.1. Percentage of Caco2shNT cells with multilumen cysts=4.5±2.5%, no lumen cysts=0.13±0.2%, multilayered cysts=0.9±1.7%, unshaped cysts=4.1±3.0% and reversed polarity cysts=0.2±0.2%. Percentage of Caco2shEpCAM#1 cells with multilumen cysts=28.6±4.9%, no lumen cysts=0.2±0.3%, multilayered cysts=10.9±3.0%, unshaped cysts=16.7±4.5% and reversed polarity cysts=9.6±2.2%. Percentage of Caco2shEpCAM#2 cells with multilumen cysts=26.6±8.5%, no lumen=3.0±1.1%, multilayered cysts=18.5±6.3%, unshaped cysts=15.8±1.5% and reversed polarity cysts=16.1±4.7%. Values are mean±s.d. (**b**–**e**) Confocal analysis of E-cadherin (**b**), actin (**c**), ezrin (**d**) and Crb3 (**e**) in 21 days 3D Matrigel cultures of control (*Caco2 shNT*) and EpCAM-silenced (*Caco2 shEpCAM*) cells. (**b**(c)) Arrowheads point toward weakened E-cadherin-based cell adhesions. (**b**(d)) Arrowheads point toward basal redirection of lateral E-cadherin. (**d**–**e**) Arrowheads point toward apical marker mislocalization on the outer side of the cysts, reflecting the loss of apico-basal polarity in shEpCAM cells. Nuclei were detected with Hoechst 33342 staining (blue). Scale bars, 10 μm. (**f**,**g**) Confocal analysis of double immunostainings for villin (green) and ZO-1 (red) (**f**), or E-cadherin (green) and ezrin (red) (**g**) in 21 days 3D Matrigel cultures of control (*Caco2 shNT*) and EpCAM-silenced (*Caco2 shEpCAM*) cells. Higher magnifications on EADs are presented in the lower panels. Nuclei were detected with Hoechst 33342 staining (blue). Scale bars, 20 μm.

**Figure 8:**
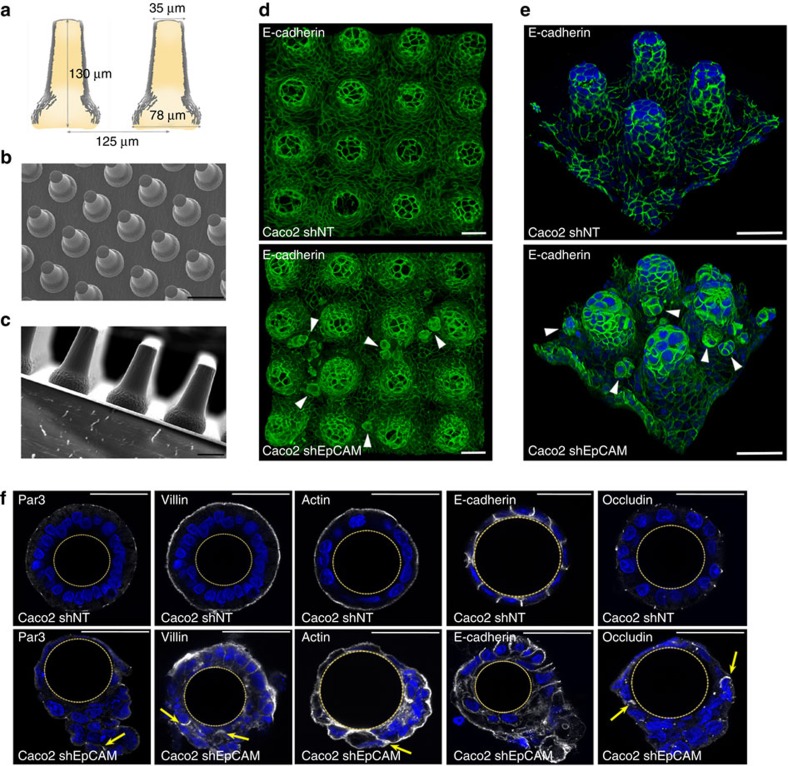
Generation of PDMS culture inserts that mimic villus topography, and highlighting of tuft-like structures in cultures EpCAM-silenced on synthetic villi. (**a**) Schematic representation of the synthetic polydimethylsiloxane (PDMS) villus inserts. (**b**,**c**) Scanning electron microscopy analyses of villus PDMS culture inserts: top (**b**) and side (**c**) views are presented. Scale bars, 50 μm (**b**) and 100 μm (**c**). (**d**,**e**) Confocal microscopy analysis of E-cadherin distribution in control (*Caco2 shNT*, upper panels) and EpCAM-silenced (*Caco2 shEpCAM*, lower panels) cells that were grown on 3D villus-like micropatterned PDMS inserts for 21 days. After z-stack acquisitions, 3D rendering was generated. White arrowheads point on tuft-like structures. Scale bars, 50 μm. (**f**) Confocal microscopy analysis of Par3, villin, actin, E-cadherin and occludin distribution in control (*Caco2 shNT*) and EpCAM-silenced (*Caco2 shEpCAM*) cells that were grown on villous PDMS inserts for 21 days. Transversal *xy* views are presented. Par3, villin, actin and occludin display abnormal lateral membrane localization in the absence of EpCAM (yellow arrows). Scale bars, 50 μm.

**Figure 9:**
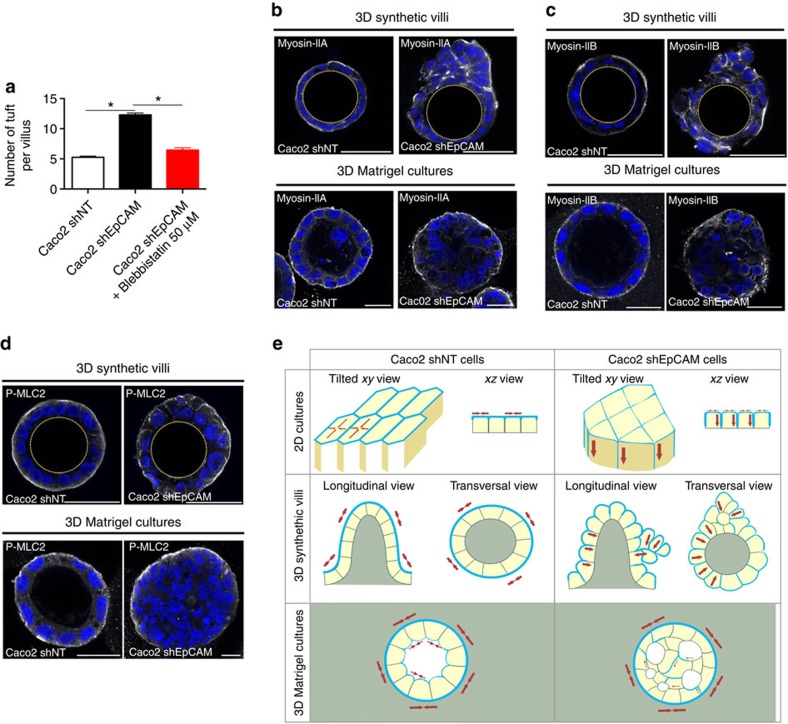
Cell contractility, and epithelial and substrate topography participate in tuft formation in the absence of EpCAM. (**a**) Statistical analysis of the number of tuft-like structures detected along the 3D microfabricated villus structures in control (*Caco2 shNT*), EpCAM-depleted cells (*Caco2 shEpCAM*) and 2h- blebbistatin-treated EpCAM-depleted cells (*Caco2 shEpCAM#1+Blebbistatin 50* μ*M*). Three independent replicates have been performed. One-way analysis of variance with unpaired *t*-test, *P<0.0001. *n*_(Caco2 shNT)_=196 villi, *n*_(Caco2 shEpCAM)_=210 villi, *n*_(Caco2 shEpCAM+Blebistatin)_=210 villi. Caco2 shNT(mean number of tufts per villus)=5, Caco2 shEpCAM(mean number of tufts per villus)=12, Caco2 shEpCAM+Blebbistatin 50 μM(mean number of tufts per villus)=6. (**b**–**d**) Confocal microscopy analysis of myosin-IIa (**b**), myosin-IIb (**c**) and P-MLC2 (**d**) distribution in control (*Caco2 shNT*) and EpCAM-silenced (*Caco2 shEpCAM*) cells that were grown on villous PDMS inserts or in 3D Matrigel cultures for 21 days. Transversal *xy* views are presented. Scale bars, 50 μm. (**e**) Schemes recapitulating the cellular and epithelial phenotypes observed in control (*Caco2 shNT*) or EpCAM-depleted (*Caco2 shEpCAM*) conditions when cells are grown in 2D, 3D synthetic villi or 3D Matrigel cultures. Contractile apparatus (blue) and predictated tensile forces (red arrows) are presented.

At the tissue level, modifications of the contractile properties at TCs in the absence of EpCAM may be at the origin of the development of tufts in CTE patients. Since tuft-like structures were not visualized in conventional two-dimensional (2D) cultures, we hypothesized that this may be attributed to the topological properties of the cell environments. Along this line, we first tested 3D Matrigel cultures. Under such conditions, classical cyst organization was disrupted in EpCAM-silenced cells, with increased amounts of multi-layered, unshaped and/or multilumenal cysts (Fig. 7a). Moreover, weakening of E-cadherin-based cell adhesion was highlighted by adhesion fractures (Fig. 7b(b,c)), as well as enrichment of lateral E-cadherin at basal sides (Fig. 7b(d), white arrowheads). Supranumerous lumens contained the microvillous components actin and ezrin, as well as the apical polarity player Crb3 (Fig. 7c–e). Interestingly, we observed E-cadherin enrichments at the most basal part of the lateral membranes (Fig. 7b(d)), as well as ectopic appearance of apical hallmarks along basal membranes (Fig. 7c–e, white arrowheads). Global apico-basal polarity was thus compromised in mutant cells, with the appearance of inverted polarity cysts (Fig. 7a). Moreover, double immunostainings for villin and ZO-1, or E-cadherin and ezrin revealed that apical domain engulfments took place from central lumens in absence of EpCAM (Fig. 7f,g). Even though these data demonstrate that collective epithelial organization in 3D is impaired on EpCAM loss of function, tuft-like structures were not detected in 3D Matrigel cysts. We hypothesized that these cultures might not be appropriate to fully reproduce intestinal epithelial behaviour. We therefore used photolithography approaches to generate 3D elastomeric substrates that mimic villous physical constraints^34^ (Fig. 8a–c). When grown on synthetic villi, control cells formed regular monolayers (Fig. 8d,e). In contrast, EpCAM-deprived cells displayed frequent tuft-like structures (Fig. 8d,e). Moreover, the absence of EpCAM caused a drastic perturbation of hallmark proteins controlling epithelial organization, including Par3, villin, actin, E-cadherin and occludin, with the frequent appearance of apical domains engulfments (Fig. 8f, yellow arrows). These results demonstrate that EpCAM-deprived cells cultured on 3D synthetic villi can phenocopy CTE cellular and tissue defects. Remarkably, treatment with the cell contractility inhibitor blebbistatin caused the concomitant disappearance of tuft-like structures and recovery of a polarized monolayer undistinguishable from controls (Fig. 9a). This result demonstrates that local hyperactivation of the contractile apparatus was at the origin of the tuft formation.

Finally, we asked why the defects of the contractile apparatus were materialized by tuft formation in 3D synthetic villus cultures, but not in 2D cultures nor in 3D Matrigel cultures. One explanation could be the differences of epithelial topology in these cell culture systems. Indeed, apical-sides are located outside in 3D synthetic villi, but inside in 3D Matrigel cultures. To test this point, we performed hanging drop cultures to generate spheroids where apical-sides are outside^35^. Whereas regular spheroids were formed in 97% of control cells, 97% of the spheroids were irregularly shaped and displayed tuft-like cell assemblies on the apical-side in the absence of EpCAM (Supplementary Fig. 10). These data show that an epithelial topology with the apical-side outside may favour tuft formation in EpCAM-mutated cells. Another explanation came from the analysis of the contractile network in the different culture systems. In 3D synthetic villi, myosin-IIa, -IIb and P-MLC2 are mainly located at the apical-side (Fig. 9b–d), as they are in 2D cultures and in human intestines (Fig. 5a,b,i,l–n, respectively). In contrast, in 3D Matrigel cultures, the contractile network is located at the basal-side facing the Matrigel, with only a small fraction detected at the apical-side (Fig. 9b–d), as previously described^36^. This suggests distinct functions of the contractile network in 3D morphogenesis according to the epithelial topology. While required for the overall cyst shaping in basal-side outside topologies^36^, it may be dedicated to the apical domain maintenance in apical-side outside topologies. On EpCAM loss, the contractile apparatus was severely disturbed in 3D synthetic villi, but kept its main distribution at the basal-side in 3D Matrigel cultures (Fig. 9b–d). Although the small proportion of myosin-IIa, -IIb and P-MLC2, normally located at the apical-side, was mislocalized in absence of EpCAM. Thus, EpCAM loss impacts the cortical contractile network when located at the apical-side, but not at the basal-side. Altogether, these results demonstrate that epithelial tufts associated with EpCAM loss-of-function originate from defects in the spatial organization of the contractile properties in the monolayer, and result in perturbation of cellular arrangements that emerge only in the particular 3D intestinal tissue environment.

## Discussion

Mechanical modulation of an epithelial layer has been reported to ensure cell adhesion, cell migration and more recently epithelial homoeostasis^37^,^38^,^39^,^40^. A direct correlation between cell contractility and front-to-back polarity in migrating cells has been shown^41^, highlighting a close relationship between polarity determinants and actomyosin activity. In *Drosophila*, deprivation of PTEN, a polarity master regulator in epithelia, caused defects in polygonal cell shape and heterogeneous distribution of the actomyosin apparatus, similarly to EpCAM silencing. However, no connection was made with the state of apico-basal epithelial organization at that time^42^. To our knowledge, few reports made a link between spatial regulation of epithelial contractile properties and apico-basal organization. Takeichi and colleagues^43^ showed that Par3 inhibits ROCK by recruiting aPKC and Par6 to the zonula adherens. In addition, aPKC inhibits the junctional actin assembly through the action of Lulu2 (refs 44, 45).

Importantly, our study highlights an intriguing correlation between the homoeostasis of the actomyosin apparatus, the cohesion of the understudied TCs and apico-basal organization. Actomyosin clustering and excess of cortical forces at TCs generates local deformation of tight junctions and displacement of tricellular proteins, which are easily reversed on myosin activity inhibition, thus showing an unexpected plasticity of these particular junctions. To date, very few studies analysed the importance of TCs in epithelial organization^26^,^46^,^47^. Notably, Furuse and colleagues demonstrated instability of tight junction belt and actomyosin network under tricellulin silencing^26^. Because of the specific formation of EADs at TCs (Fig. 3) and tricellulin displacement after EpCAM removal (Fig. 4a–d), it would have been tempting to functionally connect EpCAM and tricellulin. However, EpCAM and tricellulin do not appear to physically interact (Fig. 4g), and neither apical-to-basal distortion of the tight junction belt nor EAD formation was observed in tricellulin-silenced cells in our hands (Fig. 4h–m). Tricellulin displacement at TCs could passively follow the tight junction distortion, since tricellulin interacts with the Cdc42 GEF Tuba, a ZO-1-binding protein^48^.

Expansion of apical membrane and ectopic appearance of the brush border at TCs in EpCAM-mutated cells explain chronic intestinal absorption incapacity and pathological diarrhoea in CTE patients. The marked perturbation of the brush border absorptive structure on EpCAM silencing clearly explains the constitutional inability of CTE patients to absorb any luminal nutrients. Concomitantly, defective cell–cell interactions occur in EpCAM-mutated enterocytes. A decrease of transepithelial resistance has been demonstrated in EpCAM-silenced human intestinal T84 cells and EpCAM mutant mice^19^,^20^. Our analyses show weakened E-cadherin-based cell–cell adhesion, with the appearance of cell adhesion fractures (Fig. 1e; Supplementary Fig. 1A; Fig. 2b,d,e). In addition, claudin-7 mislocalization to TCs and expression level decrease occurs in EpCAM-deprived cells (Figs 1e and 2h, Supplementary Fig. 2) and EpCAM mutant mice, as previously reported^19^. Claudin-7 association with tight junctions remains controversial^18^, but claudin-7 loss causes incorrect paracellular barrier permeability^49^,^50^. Moreover, the apical-to-basal distortion of tight junctions at tricellular corners and TC mislocalization strongly suggest that tight junction belt integrity may be compromised. Accordingly, freeze-fracture electron microscopy analyses revealed less-organized tight junction strands in the intestinal epithelium of EpCAM mutant mice^19^. Epithelial permeability induced by this large panel of cell adhesion abnormalities could well explain the occurrence of continuous liquid leaks that correspond clinically to chronic diarrhea persisting at bowel rest in CTE patients^4^. Nevertheless, such barrier defects would also be expected to favour an increased mucosal inflammation in response to permeability to pathogens. Conversely, though very low inflammation has been described in a few early human intestinal biopsies, epithelial inflammation is far from being part of the histological characteristics observed in CTE patients. Filling of intercellular spaces with biological material such as ectopic microvilli might constitute a physical obstacle to progression of pathogens, preventing such inflammatory evolution. Altogether, epithelial and cellular defects reported in our study contribute to explain clinical symptoms observed in CTE patients.

Alteration of epithelial contractile patterning is correlated to the development of tissue lesions characteristic of a rare human congenital epithelial disorder. Tuft-like structures only appear on EpCAM-deprived synthetic villi, testifying of an enhanced mechanical stress provided by the particular topography of the monolayer substrate. We assume that different parameters may account for tuft formation: aberrant TCs, actomyosin tension distribution, epithelial organization and substrate topography (Fig. 9e). Tufts could be the result of the abnormal presence of apical domains at TCs and defective cell arrangements. Cortical forces repartition at multicellular contacts may be critical for epithelial monolayer behaviour on tissue topography, and tufts could be caused by exacerbated distortions of TCs on 3D substrates on EpCAM silencing. Moreover, in control cells cultured in 2D or 3D synthetic villus cultures, cortical tensile forces are homogeneously distributed tangentially to the tissue plane and allow for the maintenance of the monolayer cohesion^51^. EpCAM loss leads to a net modification of the actomyosin distribution and consequent contractile forces organization. Inward-pulling forces may now be locally generated at TCs, perpendicular to the 2D substrate plane. An epithelial instability is revealed in 3D synthetic villi. The geometry of 3D synthetic villi causes changes in the distribution of inward-pulling forces, which are now radially oriented with the substrate. Their convergence may provoke monolayer bending in 3D synthetic villi^33^, and may result in cell displacement apically from the monolayer, in a similar manner to what occurs during cell extrusion^52^,^53^. Tight junctions and actomyosin apparatus relocation takes place along lateral membranes during cell extrusion^52^, whereas EpCAM silencing causes their displacement solely along TCs (Fig. 2h–j; Fig. 3e). We hypothesize that EpCAM-deprived cells could undergo some form of incomplete cell extrusion and be blocked in a ‘pre-extruded state', thus producing the formation of tuft structures.

In addition, differences observed between 2D cultures and synthetic villi may be attributed to local changes of the curvature in the latter. Tuft formation takes place along the villus, and the particular shape of the villus *per se* may also account for these morphological defects. Indeed, curvature is not homogeneous when progressing from the bottom to the top of the 3D substrates since the radius varies from 75 (villus bottom) to 35 μm (villus top). Cell contractility has been reported to depend on substrate-induced curvature during epithelial closure for gaps of similar dimensions^54^. Along this line, villus topography may induce changes in the cortical contractility along the orthoradial direction from the bottom to the top, whereas cortical contractility remains isotropic on 2D substrates. We thus speculate that these changes of cell contractility may also promote tuft formation in absence of EpCAM, and further studies including various villus dimensions may help to clarify this point.

Tissue-scale changes also took place when EpCAM-silenced cells were grown in 3D Matrigel system, although of a different nature. Supranumerous lumens were formed and likely as a result of the mislocalization of tight junctions and of minor pool of apical actomyosin in the intrinsic physical confinement of cysts (Fig. 7). However, since the actomyosin majority at basal-side was not obviously perturbed on EpCAM loss, it still allowed cysts to keep a correct shape, and no tuft-structure was noticed. How is the contractile apparatus recruited either to the apical- or to the basal-side? Myosin distribution and epithelial topology seem to be intermingled, which suggest a physical modulation of myosin patterning and/or activation dictated by lumen positioning and substrate curvature at the tissue scale.

EpCAM increasingly appears as a key factor in tissue organization^14^,^15^. Its molecular mode of action at cell adhesion sites might be indirect and could in part go through actomyosin network at lateral membranes. Interestingly, EpCAM has been proposed to regulate bulk organization of the actin cytoskeleton in epithelial thymic cells^11^. Recently, myosin-II activity has been reported to be indirectly controlled by EpCAM in Xenopus epidermis and in non-polarized Caco2 cells, where the absence of EpCAM would lead to exacerbated cell contractility^15^. Our data revealed that, in fully differentiated human enterocytes, stable deprivation of EpCAM triggers global contractile activity reduction at cellular level, but leads to reshaping of actomyosin apparatus and local tricellular activation of myosin-II at subcellular level. In addition, the heterogeneity in the distribution of tensile forces according to the culture system on EpCAM loss suggests that its modulation of the actomyosin homoeostasis may be spatially restricted to the apical cortex (Fig. 9). Actomyosin network association with AJs is considered crucial for their formation and vice versa, and tension generation at lateral membranes has been reported to be required for junction disassembly^27^,^28^. Moreover, actomyosin distribution and activity are directly modulated by tight junction proteins, and silencing of ZO-1 in MDCK cells increases actomyosin association with AJs and the level of contractility generated^55^,^56^. Taking published data into account, three scenarios could be considered for EpCAM function in actomyosin homoeostasis at lateral membranes. Absence of EpCAM could impact E-cadherin complexes and may consequently destabilize the actomyosin network. Conversely, loss of EpCAM may impair homoeostasis of the actomyosin apparatus and impacts AJ integrity. Alternatively, tight junction belt disturbance may also account for actomyosin perturbation. Using high-resolution microscopy, Peifer and colleagues^56^ demonstrated that actin cables anchor at TCs. Contrary to tight junction loss in MDCK cells^56^, increased tight junction protein levels together with tight junction remodelling may have provoked actomyosin destabilization at AJs and its subsequent accumulation and activation at TCs. However, knowledge of the spatial activation of myosin-II and related kinases is fragmented, and distribution of the kinases responsible for myosin-II activation, taking into account the geometry of a columnar epithelial cell, remains to be clearly established.

**Figure 10:**
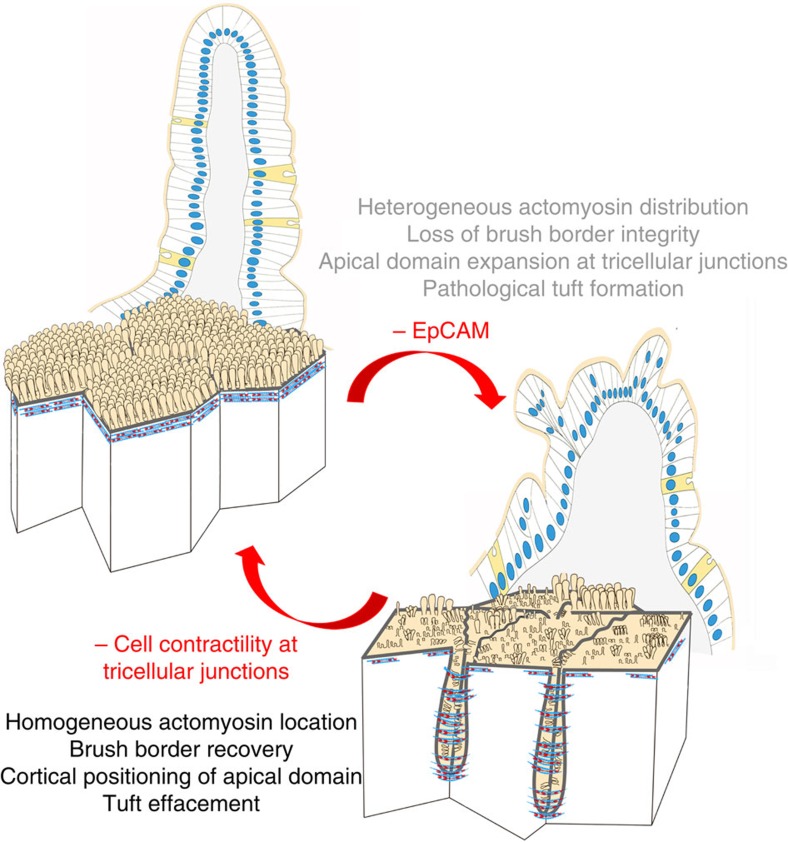
Schemes showing the effects of EpCAM deprivation and the reverse effects obtained by inhibition of the local hypercontractility using myosin-II inhibitors. Cellular distribution of actin (blue) and myosin-II (red) network is shown.

Our data support a requirement for EpCAM in the regulation of tension homeostasis within epithelial monolayer. EpCAM loss leads to a general decrease of MLC phosphorylation within fully differentiated epithelial cells, but triggers an unusual local activation of cortical myosin-II at TCs. Thus, the proper balance of cortical tension within epithelial tissues provides the link between tissue integrity and intercellular adhesion. Since cellular defects and tissue-scale changes are reversible under cell contractility inhibition, this study may open therapeutic perspectives for CTE patients (Fig. 10).

## Methods

### Human biopsies and preparation of tissue samples

Tissue samples of *EPCAM* mutated CTE children were provided by Necker-Enfants Malades, Robert Debré and Trousseau hospitals (Paris, France) and were collected either from the Necker Paediatric Anatomo-Pathology Department for retrospective analyses, or from the Gastroenterological functional exploration Departments for prospective studies. Genetic characterization of the CTE patients included here had been conducted in a previous study (Salomon *et al*.^7^). The patient cohort analysed here comprised *N*=6 CTE patients mutated for *EPCAM* and aged 4 months to 17 years, as well as *N*=12 controls (non CTE patients: *N*=2 patients with Crohn's disease, *N*=1 patient with hyperinsulinemia, *N*=1 patient with duodenal atresia, *N*=2 patients with auto-immune enteropathies, *N*=2 patients with gastro-oesophageal reflux, and *N*=4 patients with coeliac disease) aged 9 months to 14 years, who underwent duodenal endoscopy for routine explorations. The CTE patients displayed typical CTE epithelial abnormalities in intestinal biopsies and parenteral nutrition dependency, and presented *EPCAM* loss-of-function mutations. Duodenal biopsies were collected during endoscopic procedures for diagnosis and/or monitoring of CTE and control infants. All parents signed informed consent forms approved by our local ethics committee for DNA and biopsy exploitation (Unité de Recherche Clinique (URC) of Necker Hospital, URC).

### Tissue sample preparation

*N*_(Control)_=12 biopsies and *N*_(CTE)_=6 biopsies were prepared for immunohistochemical analyses. Biopsies were fixed for 2 h in either 4% formaldehyde or by successive 1 h incubations in cold 70, 90 or 100% methanol solutions and then stored at −20 °C in methanol. The samples were then paraffin embedded. A measure of 5 μm sections were de-waxed in a xylene bath, rehydrated in isopropanol and in solutions with decreasing ethanol concentrations, and were processed for either immunohistochemistry or immunostaining. For immunohistological analyses, classical hematoxylin/eosin staining procedure was used. For immunostaining, de-waxed tissue sections were blocked in 1.5% donkey serum (Sigma-Aldrich, St Louis, Missouri, USA) for 1 h. Primary antibody incubations were performed at 4 °C overnight and secondary antibody incubations at room temperature for 2 h, both in 1.5% donkey serum solution. Hoechst 33342 staining (Life Technologies, Paisley, UK) was used to detect nuclei. Tissue sections were mounted in home-made Mowiol 488 solution.

### Cell cultures

Caco2_BBE_ cells were kindly provided by Dr S. Robine (Curie Institute, Paris). Cells were routinely grown in DMEM 4.5 g l^−1^ glucose, 20% fetal bovine serum, 10% penicillin–streptomycin (Gibco, Thermo Fischer Scientific, Waltham, MA, USA). The culture medium was renewed every day. Fully polarized cells were obtained after 21 days. For 3D cultures in Matrigel, trypsinized Caco2 cells were resuspended in pure Matrigel (BD Biosciences, Franklin Lakes, NJ, USA) at a final concentration of 2.10^4^ cells ml^−1^. A measure of 30 μl of Matrigel cell suspensions were laid onto precooled 12 mm coverslips. Caco2 cysts were grown for at least 2 weeks and medium was daily renewed.

EpCAM reduction was carried out by lentiviral delivery of shRNA constructs directed against human *EPCAM* (*shEpCAM#1*: TRCN0000073734 5′-CCGGGCCGTAAACTGCTTTGTGAATCTCGAGATTCACAAAGCAGTTTACGGCTTTTTG-3′, and *shEpCAM#2* : TRCN0000073737 5′-CCGGCGCGTTAT CAACTGGATCCAACTCGAGTTGGATCCAGTTGATAACGCGTTTTTG-3′) designed and cloned into the lentiviral pLKO.1 puromycin resistant vector Mission shRNA lentiviral Transduction particle (Sigma Aldrich).

Tricellulin reduction was carried out by lentivral delivery of shRNA constructs directed against human *MARVELD2* (shTricellulin#1: TRCN0000072634 5′-CCGGGCTGCAATGATCTTCCTGTTTCTCGAGAAACAGGAAGATCATTGCAGCTTTTTG-3′, and shTricellulin#2: TRCN0000072636 5′-CCGGCGCAGCCATAGTCTATGTGAACTCGAGTTCACATAGACTATGGCTGCGTTTTTG-3′).

Control Caco2 clones (*shNT*) were generated using pLKO.1-puro non-target shRNA control transduction particles SHC016V (5′-CCGGGCGCGATAGCGCTAATAATTTCTCGAGAAATTATTAGCGCTATCGCGCTTTTT-3′). Clone generation was perfomed in DMEM supplemented with 20% FBS, 10% penicillin/streptomycin and 2 μg ml^−1^ puromycin (Invivogen, San Diego, CA, USA) and selected clones were routinely maintained in 1 μg ml^−1^ puromycin. Efficiency and specificity of EpCAM reduction was assessed by WBs.

shRNA-resistant EpCAM sequence (shEpCAM#1 resistant: 5′-ATGGCGCCCCCGCAGGTCCTCGCGTTCGGGCTTCTGCTTGCCGCGGCGACGGCGACTTTTGCCGCAGCTCAGGAAGAATGTGTCTGTGAAAACTACAAGCTGGCTGTGAATTGTTTCGTCAACAATAATCGTCAATGCCAGTGTACTTCAGTTGGTGCACAAAATACTGTCATTTGCTCAAAGCTGGCTGCCAAATGTTTGGTGATGAAGGCAGAAATGAATGGCTCAAAACTTGGGAGAAGAGCAAAACCTGAAGGGGCCCTCCAGAACAATGATGGGCTTTATGATCCTGACTGCGATGAGAGCGGGCTCTTTAAGGCCAAGCAGTGCAACGGCACCTCCATGTGCTGGTGTGTGAACACTGCTGGGGTCAGAAGAACAGACAAGGACACTGAAATAACCTGCTCTGAGCGAGTGAGAACCTACTGGATCATCATTGAACTAAAACACAAAGCAAGAGAAAAACCTTATGATAGTAAAAGTTTGCGGACTGCACTTCAGAAGGAGATCACAACGCGTTATCAACTGGATCCAAAATTTATCACGAGTATTTTGTATGAGAATAATGTTATCACTATTGATCTGGTTCAAAATTCTTCTCAAAAAACTCAGAATGATGTGGACATAGCTGATGTGGCTTATTATTTTGAAAAAGATGTTAAAGGTGAATCCTTGTTTCATTCTAAGAAAATGGACCTGACAGTAAATGGGGAACAACTGGATCTGGATCCTGGTCAAACTTTAATTTATTATGTTGATGAAAAAGCACCTGAATTCTCAATGCAGGGTCTAAAAGCTGGTGTTATTGCTGTTATTGTGGTTGTGGTGATAGCAGTTGTTGCTGGAATTGTTGTGCTGGTTATTTCCAGAAAGAAGAGAATGGCAAAGTATGAGAAGGCTGAGATAAAGGAGATGGGTGAGATGCATAGGGAACTCAATGCATAA-3′) was provided by Invitrogen and cloned into a pEGFP-N1 backbone using the following primers (Eurofins genomics): Forward: 5′-aattctgcagtcgacggtaccATGGCGCCCCCGCAGGTC-3′, Reverse: 5′-caccatggtggc gaccaggtggatcccgggTGCATTGAGTTCCCTATGCATCTCA-3′. shEpCAM#1-resistant Caco-2 clones were generated by transfection with Lipofectamine 2000 (Sigma Aldrich) and selection in DMEM supplemented with 20% FBS, 10% penicillin/streptomycin, 2 μg ml^−1^ puromycin and 0.5 mg ml^−1^ geneticin (Life Technologies).

Hanging drop cultures were used to generate 3D spheroids^57^. A measure of 10 μl drops of single cell suspension (concentration 2 × 10^6^ cells ml^−1^) were deposited on the inverted lid of a 60 mm culture dish. The lid was placed back, and 5 ml of phosphate-buffered saline (PBS) was placed in the bottom of the dish to create a wet chamber. After 48 h, spheroids were imaged using Keyence VHX 2000E microscope.

### Western blots

WBs were performed as follows. Cells were lysed for 30 min with lysis buffer, that is, 50 mM Tris per HCl pH 8.0, 150 mM NaCl, 1 mM DTT, 0.5% NP-40, 2 mM EGTA, 1 mM sodium orthovanadate with complete protease inhibitor cocktail (Roche, Basel, Switzerland). Insoluble debris were removed by centrifugation at 13,000*g* for 15 min and supernatants were used as cell lysates. Protein amounts were determined by Bradford assay. For each condition, 50 μg of proteins were loaded per well in Novex Tris-Glycine pre-cast gels (Thermo Fischer) and transferred on nitrocellulose membranes using iBlot Dry blotting system (Thermo Fischer). Proteins were detected with either horseradish peroxidase-linked goat anti-mouse IgG antibody (dilution 1:10,000; Sigma-Aldrich) or horseradish peroxidase-linked donkey anti-rabbit IgG antibody (dilution 1:10,000, GE Healthcare, Buckinghamshire, UK), and SuperSigna West Femto Maximum Sensitivity Substrate (Thermo Fischer Scientific) and visualized on ImageQuant LAS4000 (GE-Healthcare). Signal quantification was performed using Fiji software. Uncropped scand of WBs can be found in Supplementary Figs 11–14.

### Antibodies and reagents

Mouse monoclonal antibody directed against villin (clone ID2C3, immunofluorescence (IF) dilution 1:100), rabbit polyclonal antibody directed against ezrin (IF dilution 1:200, WB dilution 1:500) and mouse monoclonal antibody directed against lactase phlorizin hydrolase (IF dilution 1:100) were kindly provided by Dr S. Robine (Curie Institute, Paris). Mouse monoclonal antibody directed against Crb3 (IF dilution 1:100) was kindly provided by Dr A. Le Bivic (IBDM, Luminy, France). Mouse monoclonal (#AM00558PU-N, IF dilution 1:100) and rabbit polyclonal (#ab71916, IF dilution 1:100, WB dilution 1:1,000) antibodies directed against EpCAM were from Acris (Herford, Germany) and Abcam (Cambridge, MA, USA), respectively. Mouse monoclonal antibodies directed against E-cadherin (#610181, IF dilution 1:100) and aPKC (#610207 clone 41, IF dilution 1:100) were from BD Biosciences (Rockville, MD, USA). Mouse monoclonal antibody directed against pancytokeratin (#SRE0061, clone PCK-26, IF dilution 1:100) was from Sigma Aldrich. Rabbit polyclonal antibodies directed against Na^+^/K^+^-ATPase (#ab76020, IF dilution 1:100) and occludin (#TA306787, IF dilution 1:100, WB dilution 1:1,000) were from Abcam (Cambridge, UK) and Acris (Herford), respectively. Rat monoclonal antibody directed against occludin (clone MOC37, IF dilution 1:50) was a gift from Sylvie Robine. Rabbit polyclonal antibodies directed against tricellulin (#48-8400, IF dilution 1:100, WB dilution 1:1,000), ZO-1 (#40-2300, IF dilution 1:200), Par3 (#PA5-33529, IF dilution 1:100, WB dilution 1:500) and claudin-7 (#34-9100, IF dilution 1:200, WB dilution 1:1,000) were from Life Technologies. Rabbit polyclonal antibody directed against Scribble (#sc-28737, IF dilution 1:100) was from Santa Cruz Biotechnology (Santa Cruz, CA, USA). Rabbit polyclonal antibodies directed against myosin-IIA (#909801, clone Poly19098, IF dilution 1:200) and myosin-IIb (#909901, clone Poly19099, IF dilution 1:200) were from Biolegend (formely Covance, Princeton, NJ, USA). Rabbit polyclonal antibody directed against MLC2 (#3672, WB dilution 1:1,000) was from Cell Signaling (Danvers, MA, USA). Rabbit polyclonal antibody directed against P-MLC2 (S19/S20, #600-401-416, IF dilution 1:100, WB dilution 1:500) was from Rockland (Limerick, PA, USA). Rabbit polyclonal antibody directed against phosphomyosin light chain (P-MLC2; T18/S19, #3674, WB dilution 1:500) was from Cell Signaling (Danvers, MA, USA). Monoclonal antibody directed against GAPDH (#60004-1-Ig, clone 1E6D9, WB dilution 1:500) was from Proteintech (Chicago, IL, USA). Monoclonal antibody directed against GFP (#11814460001, clones 7.1 and 13.1, WB dilution 1:1,000) was from Roche (Basel, Switzerland). Anti-mouse Alexa-488 and anti-rabbit Alexa-568 secondary antibodies (dilution 1:250) were from Life Technologies. Phalloidin-FITC and phalloidin-598 were from Sigma-Aldrich and Life Technologies, respectively.

### Immunofluorescence and electron microscopy analyses

For immunofluorescence, cells grown on solid supports or on Matrigel matrix were fixed either in 4% formaldehyde for 15 min or 1 h, respectively, or in −20 °C precooled methanol for 5 or 30 min, respectively. Cell permeabilisation was perfomed by incubating the cells in 0.02% saponine (Sigma-Aldrich) in PBS, and saturation in PBS containing 0.025% saponine, 1% BSA. Primary antibody incubations were performed in PBS containing 0.025% saponine and 1% BSA at 4 °C for 12 h for 2D cultures or 24 h for Matrigel suspensions. Secondary antibodies (Life Technologies) were incubated 1 h for 2D cultures or 12 h for Matrigel suspension. Nuclei were detected by Hoechst 33342 staining (Life Technologies). Cells were mounted in Mowiol 488 solution (Calbiochem, Merck, Darmstadt, Germany).

Confocal images were acquired on a Leica TCS SP5, Leica TCS SP8 and Zeiss LSM780 microscopes with × 40 or × 63 water planapochromat lenses (Leica Microsystems, Wetzlar) and processed with ImageJ. For 3D microfabricated device imaging, SP8 acquisitions were processed for 3D rendering provided by the Leica software package.

Quantifications of P-MLC2 and MLC2 fluorescence were performed on a LEICA TCS SP8 using hybrid detector. Image stacks were acquired using counting mode setup with identical acquisition settings (laser power, objectives and magnifications) for each acquired image and condition. Fluorescence quantifications were done using Fiji software. To determine total fluorescence per cell, single cell was manually delimitated on phalloidin treated cells for cell edge determination. For each experiment set, the mean P-MLC2 fluorescence per cell was reported on the mean MLC2 fluorescence per cell. To determine fluorescence intensity at TCs versus bicellular contacts, cell edges were visualized on phalloidin treated cells. Tricellular and bicellular contacts were manually delimitated. For each cell, the total pixel intensity of TCs was reported on the total pixel intensity of bicellular contacts.

3D-SIM images were acquired on the Nikon N-SIM system using a × 100 APO TIRF objective in the NIKON Imaging Center at the Curie Institute (Paris, France). The images reconstruction based on the Gustafsson methods^58^ were performed on the NIS-Elements Ar software.

*N*_(Control)_=3 biopsies, *N*_(CTE)_=3 biopsies were used for transmission electron microscopy analyses. Biopsies were immersion-fixed in 2.5% glutaraldehyde (Electron Microscopy Sciences (EMS), Hatfield, PA, USA) in 0.1 M cacodylate buffer (pH 7.4; Sigma-Aldrich) for 2 h at 4 °C and then in 1% osmic acid (EMS) in 0.1 M cacodylate buffer for 1 h. Standard procedures for dehydration and embedding in Epon-Araldite (EMS) were used. Same procedure was used for Caco2 monolayer samples. Ultrathin sections were stained with uranyl acetate and lead citrate (Sigma-Aldrich) solutions before examination using a Tecnai T12 microscope (FEI, Eindhoven, Netherlands).

For SEM analyses, cells were fixed overnight at 4 °C in 2.5% glutaraldehyde in cacodylate buffer. They were dehydrated in a graded series of ethanol solutions, then dried by the CO_2_ critical-point method using Emcpd300 (Leica microsystems). Samples were mounted on an aluminium stub with a silver lacquer and sputter coated with a 5–10 nm platinum layer using Emace600 (Leica microsystems). Acquisitions were performed using a GeminiSEM 500 (Zeiss).

### Blebbistatin treatment

Cells were incubated in 5 or 50 μM blebbistatin (Sigma-Aldrich) in DMSO for 10 min or 2 h, then washed out with PBS and fixed for immunostaining. Cells were incubated for 2 h in DMSO as controls.

For live cell imaging, actin cytoskeleton was stained using SiR-Actin (Spirochrome, Stein am Rehein, Stwitzerland). Cells were incubated 30 min with 1 μM SiR-Actin and maintained in 100 nM SiR-Actin in cell culture medium along the image acquisitions. Before acquisitions, blebbistatine was added in culture medium at 50 μM final concentration. Time-lapse acquisitions were performed on a Leica TCS SP8, using a × 40 numerical aperture 1.3 oil objective. Images were acquired every 3 min and analysed using Fiji software.

### Biomimetic cell culture inserts and tuft quantifications

Silicon wafers were patterned with arrays of cylindrical pits that mimic intestinal villus topography using soft photolithography and deep etching process^59^. Wafers were silanized with tridecafluoro-trichlorosilane to facilitate elastomer layer release after curing. Liquid silicone prepolymer, that is, polydimethylsiloxane (PDMS; Sylgard 182+184, Dow-Corning, Auburn, MI, USA), was poured over the wafer, cured at 65 °C overnight and peeled off. After release from the mold, the PDMS patterned replica was cut into subunits for cell culture, ioxidized and sterilized in an air plasma (Harrick, Ithaca, NY, USA) for 5 min. The cells were then cultured on the PDMS patterns in Petri dishes.

For tuft quantification, shNT and shEpCAM Caco2 cells were cultured on PDMS microdevices until full polarization, then immunostained for E-cadherin and stained with Hoechst33342 for nucleus detection. Three independent experiments were used and five separate fields of 42 micropillars were analysed for each cell type. The number of tufts on each pillar was collected and compared using Dunn's multiple comparison tests (Kruskal–Wallis statistics, GraphPad Prism software, GraphPad Software, Inc.).

### Data availability

The data that support the findings of this study are available from the corresponding author on request.

## Additional information

**How to cite this article:** Solomon, J. *et al*. Contractile forces at tricellular contacts modulate epithelial organization and monolayer integrity. *Nat. Commun.* **8,** 13998 doi: 10.1038/ncomms13998 (2017).

**Publisher's note:** Springer Nature remains neutral with regard to jurisdictional claims in published maps and institutional affiliations.

## Supplementary Material

Supplementary Information Supplementary Figures
