# Supplementary material for: Contractile forces at tricellular contacts modulate epithelial organization and monolayer integrity
Source: Nat Commun. 2017 Jan 13;8:13998. doi: 10.1038/ncomms13998 (PMC5241865; doi:10.1038/ncomms13998)
Supplement: Supplementary Information — Supplementary Figures [file ncomms13998-s1.pdf]

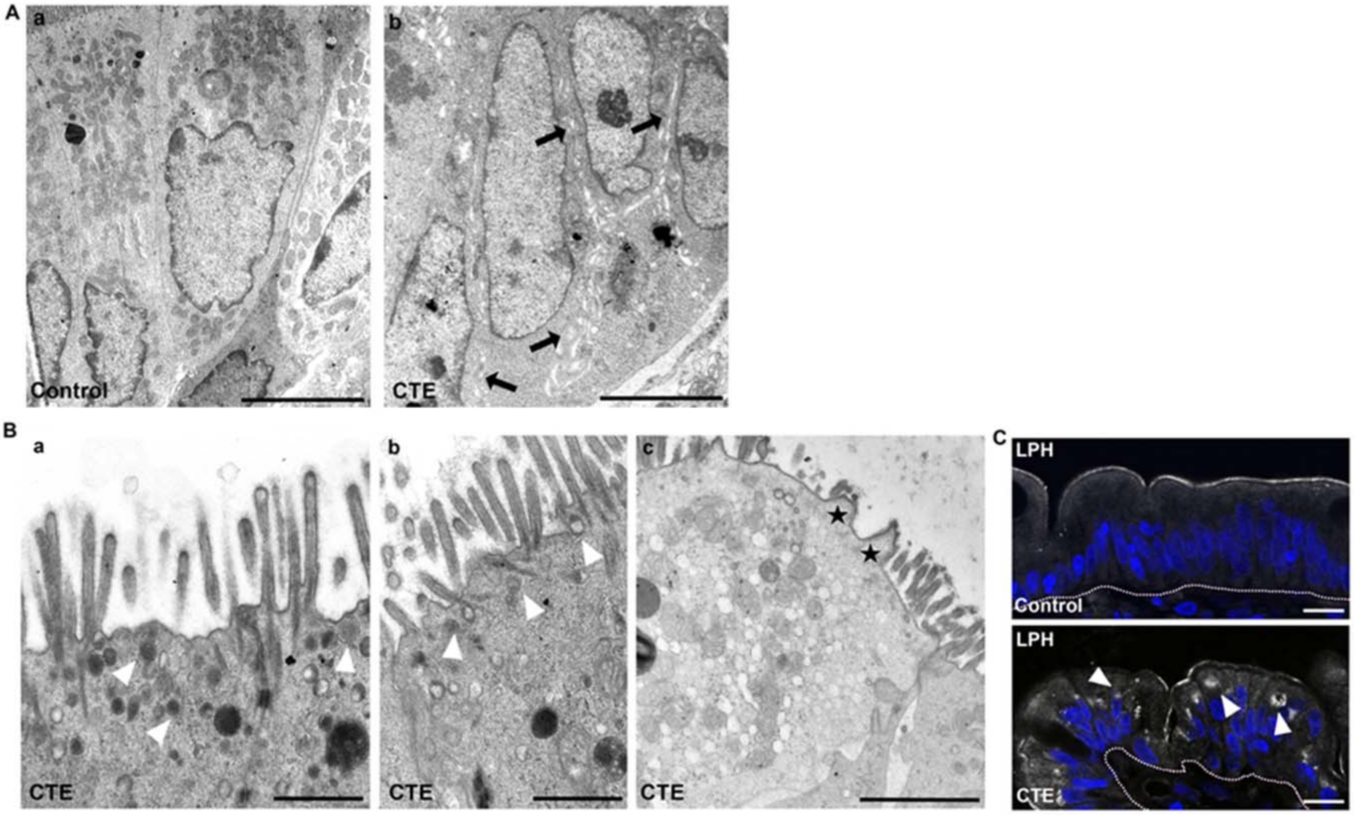

<u>Supplementary Figure 1:</u> Cell-cell adhesion and terminal web defects occur in CTE patients. (A), TEM ultrastructural analysis of lateral membranes in control (***a***) and CTE (***b***) biopsies. Arrows point at interdigitations on lateral membranes. N ($_{\text{Control}}$) = 3 biopsies, N ($_{\text{CTE}}$) = 3 biopsies. Scale bars, 5 µm. (**B)**, TEM ultrastructural analysis of enterocyte apical cortex in CTE biopsies. **Ba-b**, White arrowheads point at abnormal accumulation of electron dense vacuoles in the terminal web in CTE samples. **Bc**, Black stars show plasma membrane herniations. N ($_{\text{Control}}$) = 3 biopsies, N ($_{\text{CTE}}$) = 3 biopsies. Scale bars **Ba-b**, 1 µm; scale bar **Bc**, 2 µm. (**C**), Confocal microscopy analyses of LPH distribution after immunostaining on control (*upper panel*) or CTE (*lower panel*) biopsy paraffin sections. In CTE epithelia, LPH was accumulated in perinuclear compartments (*white arrowheads*). N ($_{\text{Control}}$) = 12 biopsies, N ($_{\text{CTE}}$) = 6 biopsies. Scale bars, 10 µm.

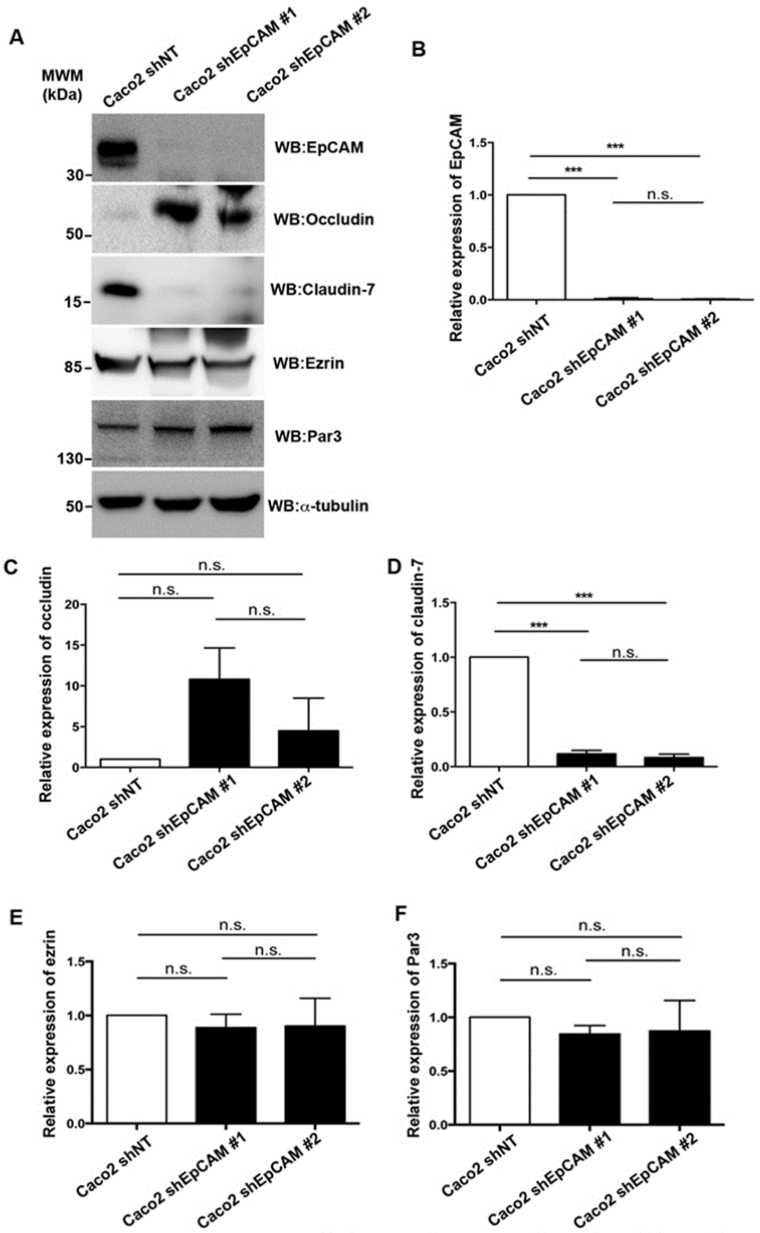

<u>Supplementary Figure 2:</u> Expression levels in EpCAM-depleted cells. (**A**) Western blot analysis of the level of the expression of EpCAM, occludin, claudin-7, ezrin and Par3 in control (*Caco2 shNT*) or EpCAM-deprived (*Caco2 shEpCAM#1* and *Caco2 shEpCAM#2*) clones. α-tubulin was used as loading control. (**B-F**) Statistical analysis of EpCAM, occludin, claudin-7, ezrin or Par3 amounts relative to loading control amounts in control (*Caco2 shNT*) or EpCAM-deprived (*Caco2 shEpCAM #1* and *Caco2 shEpCAM #2*) clones. The analysis was performed based on three independent experiments. Statistical significance was determined using one-way ANOVA and Tukey's multiple comparison tests. ***, *P*-value < 0.0001. Percentage of EpCAM expression in control cells = 100 %, in Caco2 shEpCAM#1 cells = 1.08 ± 0.0009 %, and in Caco2 shEpCAM#2 = 0.72 ± 0.003 %. Percentage of occludin expression in control cells = 100 %, in Caco2 sh EpCAM #1 cells = 1081 ±383 %, and in Caco2 sh EpCAM #2 = 449.1 ±399 %. Percentage of claudin-7 expression in control cells = 100 %, in Caco2 sh EpCAM #1 cells = 11.71 ±0.03 %, and in Caco2 sh EpCAM #2 = 8.34 ±0.03 %. Percentage of ezrin expression in control cells = 100 %, in Caco2 sh EpCAM #1 cells = 88.75 ± 0.12 %, and in Caco2 sh EpCAM #2 = 90.31 ± 0.26 %. Percentage of Par3 expression in control cells = 100 %, in Caco2 sh EpCAM #1 cells = 84.62 ± 0.08 %, and in Caco2 sh EpCAM #2 = 87.43 ± 0.28 %. Values are mean ± s.e.m.

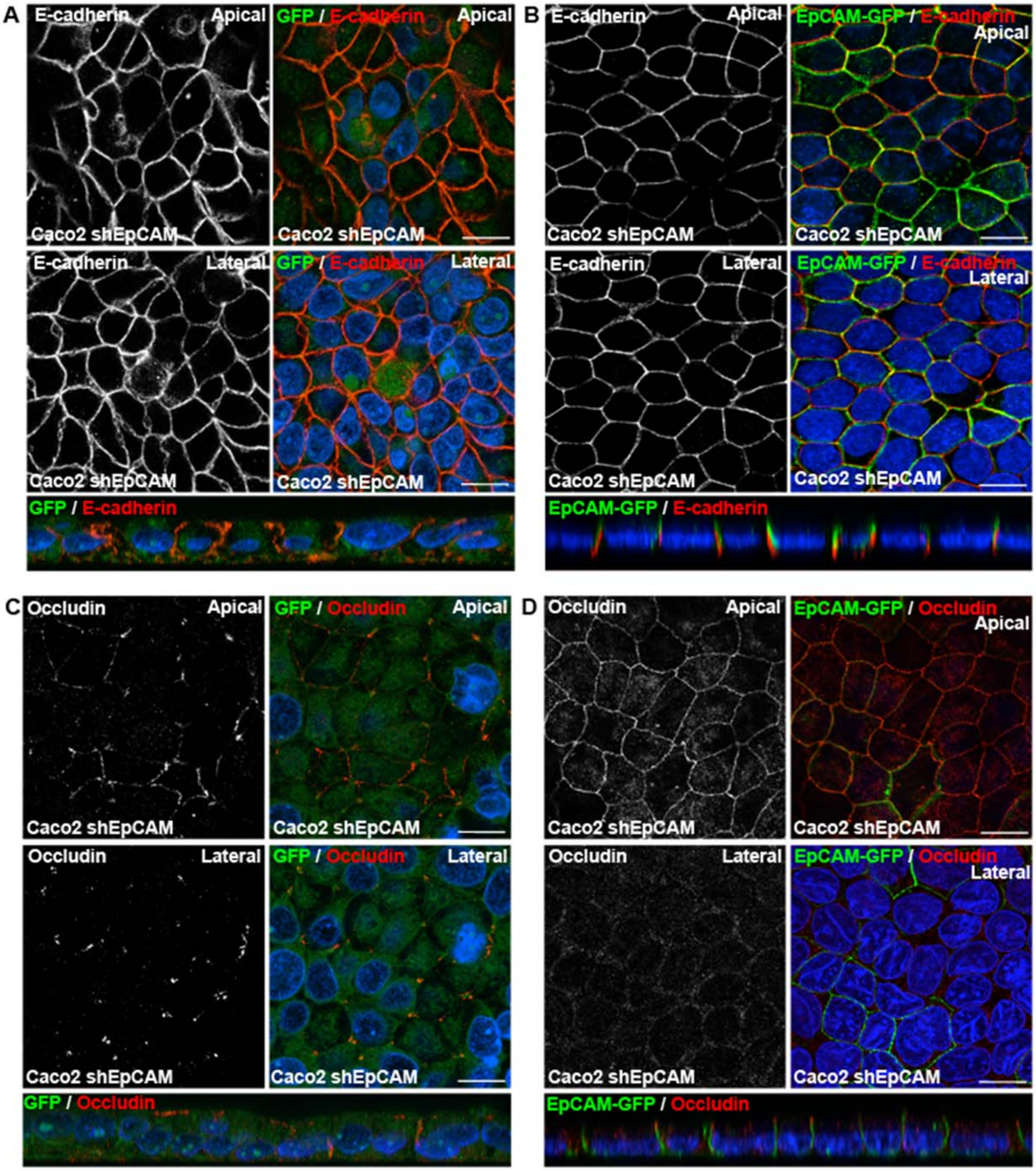

__Suppplementary Figure 3:__ **Recovering of cell-cell adhesion integrity and tight junction belt positioning after EpCAM rescue in EpCAM-mutant cells.** Confocal microscopy analysis of immunostainings for E-cadherin (*red*) (**A-B**) and occludin (*red*) (**C-D**), on the apical (*Apical*) and lateral (*Lateral*) sides in EpCAM-depleted cells transfected with plasmids coding for GFP (**A** and **C**) or shRNA resistant EpCAM-GFP (**B** and **D**) constructs. *xy* and *xz* views are presented. Scale bars, 5 µm. Nuclei were detected with Hoechst 33342 staining (*blue*).

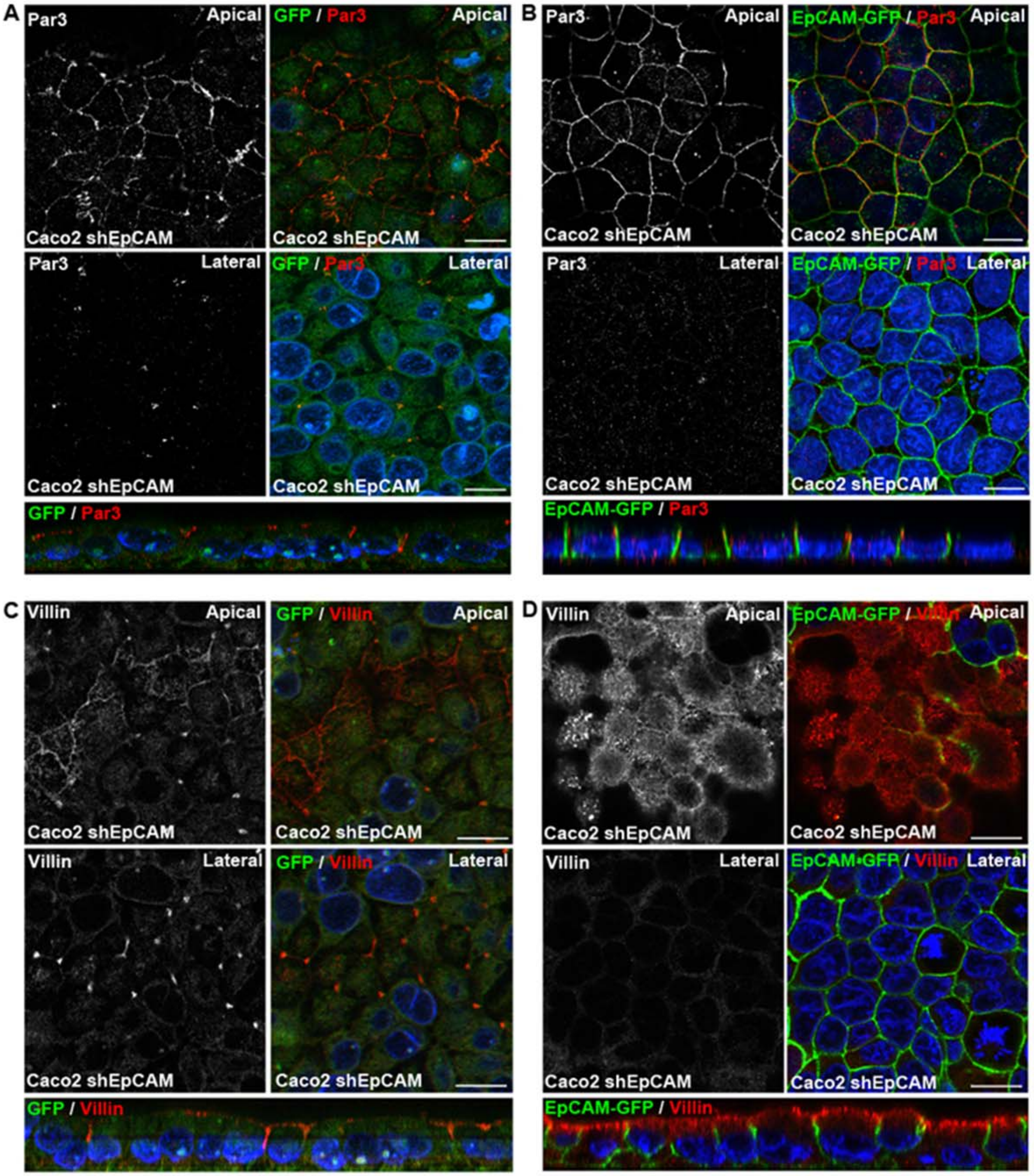

<u>Supplementary Figure 4:</u> Recovering of correct apical domain positioning after EpCAM rescue in EpCAM-mutant cells. Confocal microscopy analysis of immunostainings for Par3 (*red*) (**A-B**) and villin (*red*) (**C-D**), on the apical (*Apical*) and lateral (*Lateral*) sides in EpCAM-depleted cells transfected with plasmids coding for GFP (**A** and **C**) or shRNA resistant EpCAM-GFP (**B** and **D**) constructs. *xy* and *xz* views are presented. Scale bars, 5 µm. Nuclei were detected with Hoechst 33342 staining (*blue*).

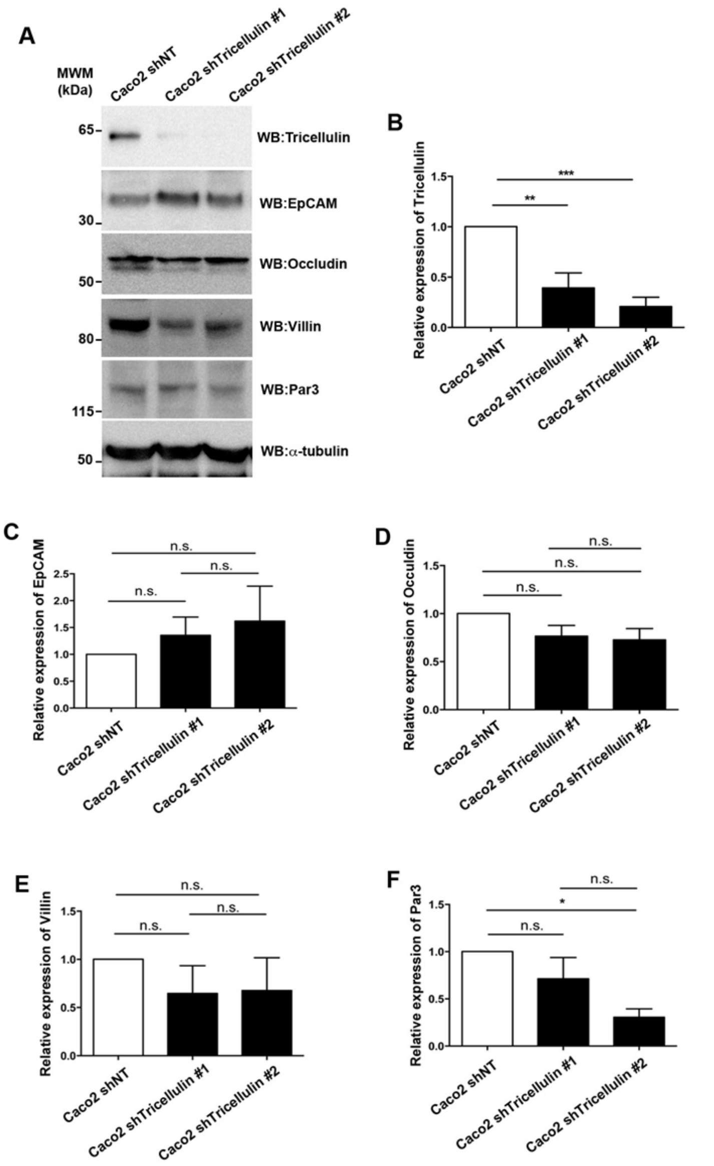

Supplementary Figure 5: Expression levels in tricellulin-depleted cells. (**A**) Western blot analysis of the level of the expression of tricellulin, EpCAM, occludin, villin and Par3 in control (*Caco2 shNT*) or tricellulin-deprived (*Caco2 shTricellulin #1* and *Caco2 shTricellulin #2*) clones. α-tubulin was used as loading control. (**B-F**) Statistical analysis of tricellulin, EpCAM, occludin, villin or Par3 amounts relative to α-tubulin amounts in control (*Caco2 shNT*) or tricellulin-deprived (*Caco2 shTricellulin #1* and *Caco2 shTricellulin #2*) clones. The analysis was performed based on three independent experiments. Statistical significance was determined using one-way ANOVA and Tukey's multiple comparison tests. *, P-value < 0.1, **, P-value < 0.01, ***, P-value < 0.001. Percentage of tricellulin expression in Caco2shNT cells = 100 %, in Caco2 shTricellulin#1 cells = 39.28 ±0.15 %, and in Caco2 shTricellulin#2 = 20.79 ±0.09 %. Percentage of EpCAM expression in Caco2shNT cells = 100 %, in Caco2 shTricellulin#1 cells = 135 ±0.34 %, and in Caco2 shTricellulin#2 = 162 ±0.65 %. Percentage of occludin expression in Caco2shNT cells = 100 %, in Caco2 shTricellulin#1 cells = 76.56 ±0.11 %, and in Caco2 shTricellulin#2 = 72.65 ±0.12 %. Percentage of villin expression in Caco2shNT cells = 100 %, in Caco2 shTricellulin#1 cells = 64.69 ±0.29 %, and in Caco2 shTricellulin#2 = 67.64 ±0.34 %. Percentage of Par3 expression in Caco2shNT cells = 100 %, in Caco2 shTricellulin#1 cells = 71.26 ±0.23 %, and in Caco2 shTricellulin#2 = 30.51 ±0.09 %. Values are mean±s.e.m.

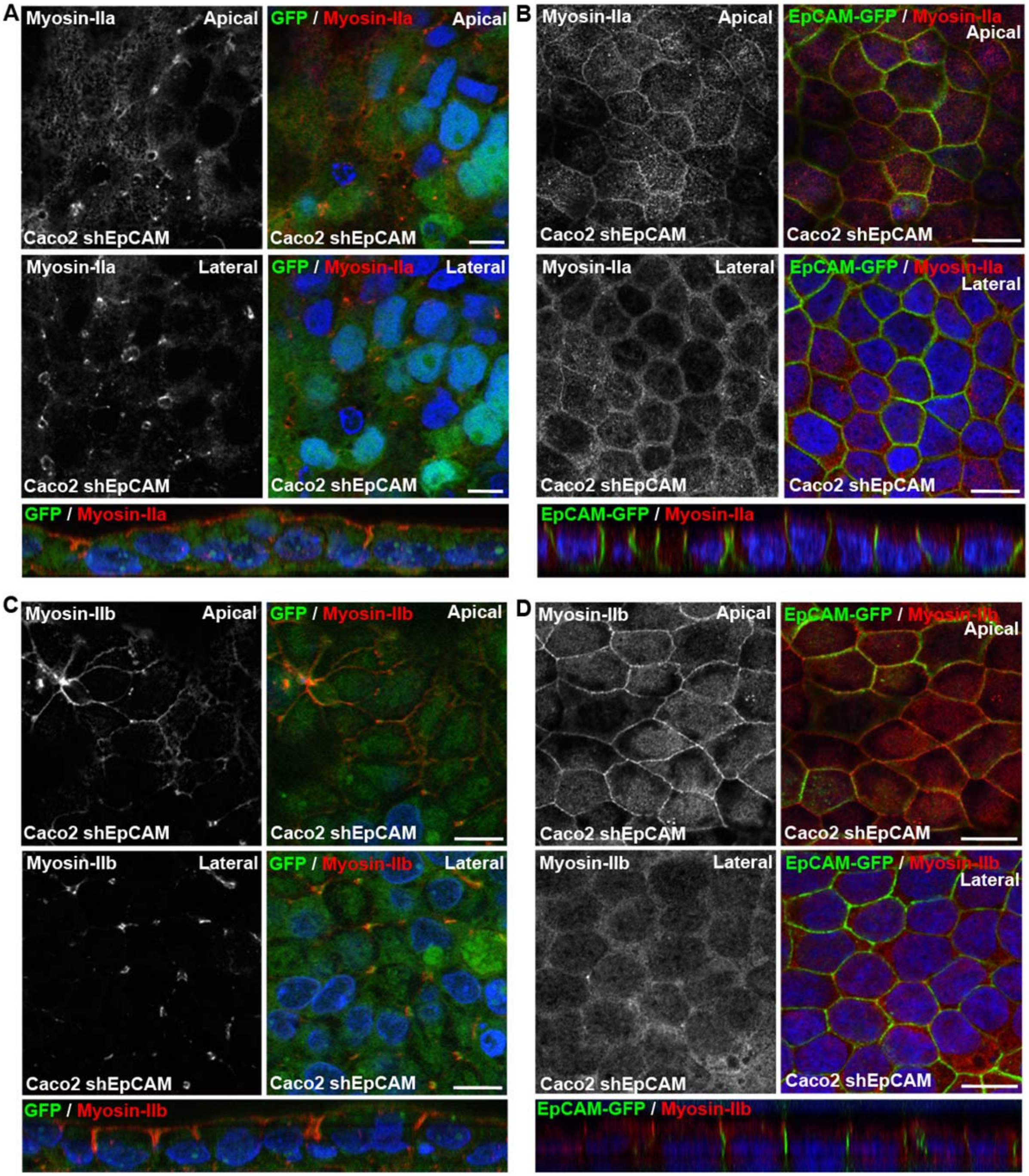

<u>Supplementary Figure 6:</u> **Recovering of correct actomyosin positioning after EpCAM rescue in EpCAM-mutant cells.** Confocal microscopy analysis of immunostainings for myosin-IIa (*red*) (**A-B**) and myosin-IIb (*red*) (**C-D**), on the apical (*Apical*) and lateral (*Lateral*) sides in EpCAM-depleted cells transfected with plasmids coding for GFP (**A** and **C**) or shRNA resistant EpCAM-GFP (**B** and **D**) constructs. *xy* and *xz* views are presented. Scale bars, 5 µm. Nuclei were detected with Hoechst 33342 staining (*blue*).

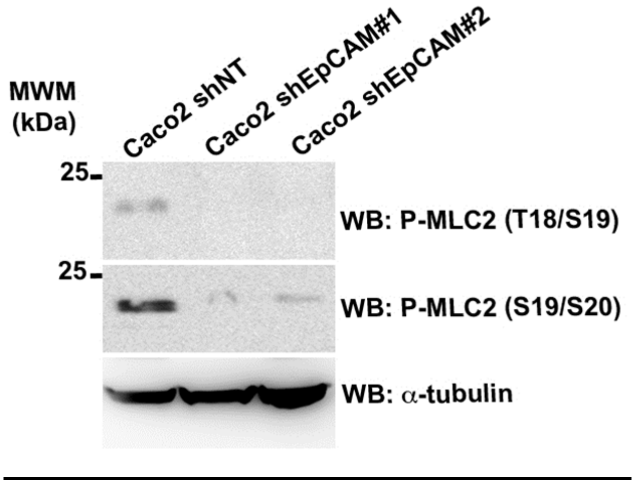

Supplementary Figure 7: **P-MLC2 amounts are decreased in EpCAM-mutant cells** Western blot analysis of P-MLC2 amounts in control (*Caco2 shNT*) or EpCAM-depleted (*Caco2 shEpCAM#1* and *Caco2 shEpCAM#2*) cells, using antibodies directed against either P-MLC2 (T18/S19) or P-MLC2 (S19/S20). α-tubulin was used as loading control.

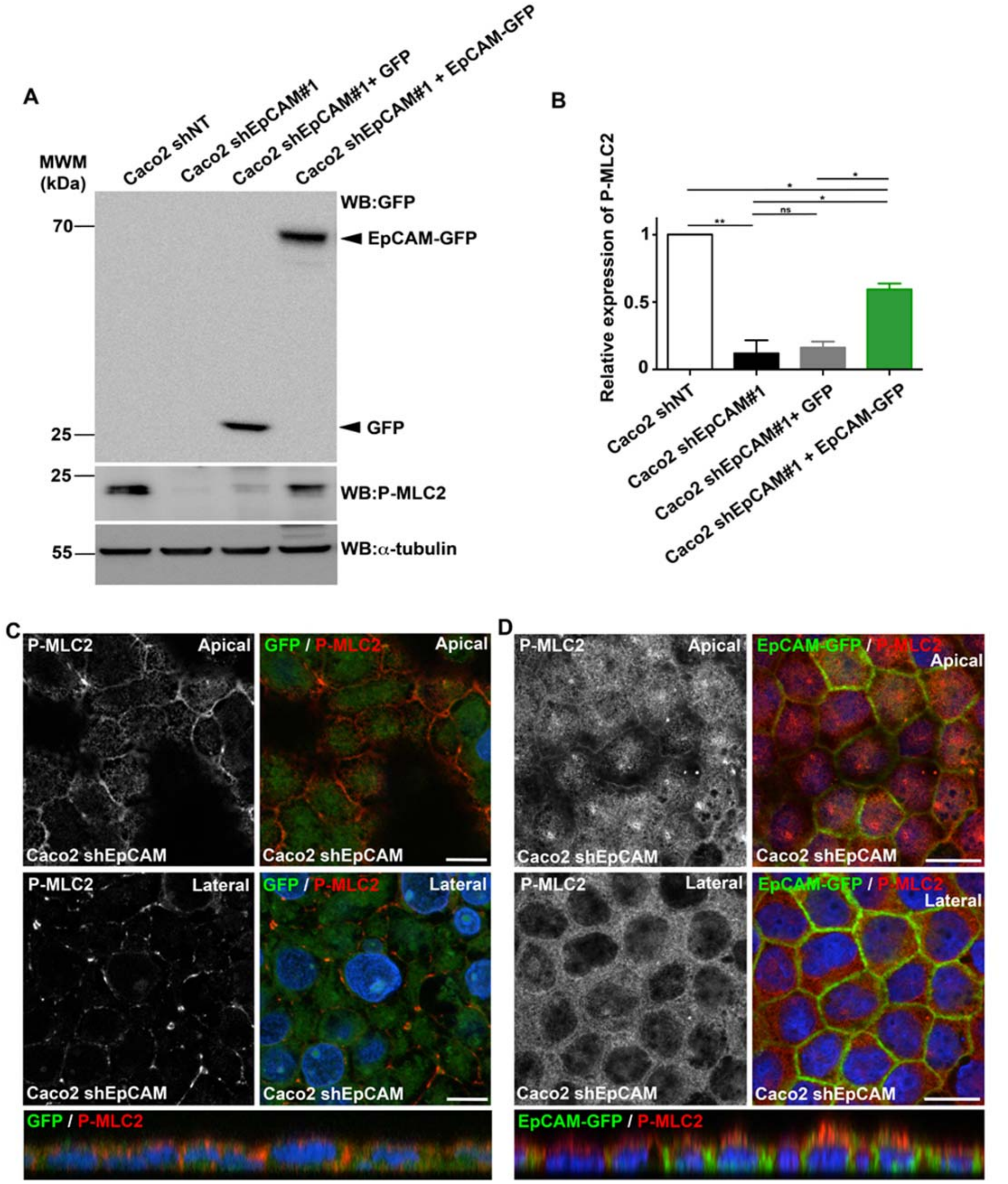

Supplementary Figure 8: **Restoration of proper P-MLC2 amounts and distribution after EpCAM rescue in EpCAM-mutant cells.** (**A**) Western blot analysis of P-MLC2 amounts in control (*Caco2 shNT*) or EpCAM-depleted (*Caco2 shEpCAM#1*) cells, transfected either with GFP (*Caco2 shEpCAM#1+GFP*) or EpCAM-GFP (*Caco2 shEpCAM#1+EpCAM-GFP*) constructs. **(B)** Statistical significance was determined using one-way ANOVA and Tukey's multiple comparison tests. The analysis was performed based on three independent experiments. *, *P*-value < 0.1, **, *P*-value < 0.01. Percentage of P-MLC2 expression in Caco2shNT cells = 100 %, in Caco2 shEpCAM#1 cells = 11.89 ±9.74 %, in Caco2 shEpCAM#1+GFP = 16.21 ±4.45 % and in Caco2 shEpCAM#1+EpCAM-GFP = 59.5±4.34 %. Values are mean±s.e.m. **(C-D)** Confocal microscopy analysis of immunostainings for P-MLC2 (*red*) on the apical (*Apical*) and lateral (*Lateral*) sides in EpCAM-depleted cells transfected with plasmids coding for GFP (**C**) or shRNA resistant EpCAM-GFP (**D**) constructs. *xy* and *xz* views are presented. Scale bars, 5 µm. Nuclei were detected with Hoechst 33342 staining (*blue*).

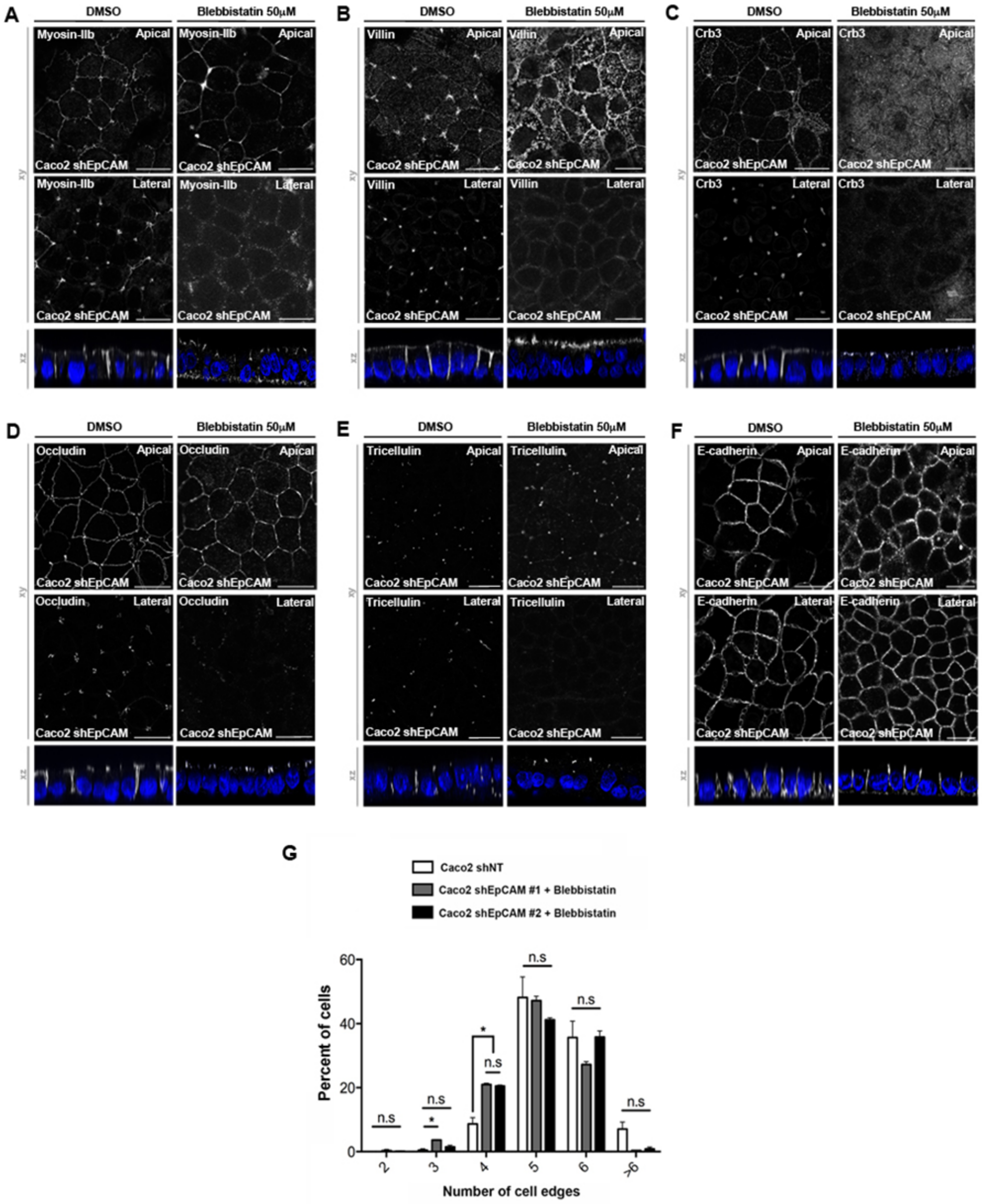

Supplementary Figure 9: Recovery of correct epithelial organization upon blebbistatin treatment. (**A-F**) Confocal microscopy analysis of myosin-IIb (**A**), villin (**B**), Crb3 (**C**), occludin (**D**), tricellulin (**E**) and E-cadherin (**F**) localization in Caco2 shEpCAM cells without or with 50 µM blebbistatin treatment for 2 h. Scale bars, 5 µm. (**G**) Statistical analysis of polygonal shape in control (*Caco2 shNT*, *white*) and 2h 50 µM blebbistatin-treated EpCAM-deprived cells (*Caco2 shEpCAM#1 + blebbistatin*, *gray*, and *Caco2 shEpCAM#2 + blebbistatin*, *black*). Three independent experiments were performed. N ($_{\text{Caco2shNT}}$) = 517 cells, N ($_{\text{Caco2shEpCAM\#1+blebbistatin}}$) = 732 and N ($_{\text{Caco2shEpCAM\#2+blebbistatin}}$) = 688. Percentage of Caco2 shNT cells with 2 cell edges = 0 %, with 3 cell edges = 0,455±0,455 %, with 4 cell edges = 8,673±1,967 %, with 5 cell edges = 48,154±6,422 %, with 6 cell edges = 35,652±5,131 %, with more than 6 cell edges = 7,067±2,224 %. Percentage of Caco2 shEpCAM#1 cells with 2 cell edges = 0,391±0,391 %, with 3 cell edges = 3,697±0,129 %, with 4 cell edges = 21,010±0,419 %, with 5 cell edges = 47,228±1,419 %, with 6 cell edges = 27,263±0,913 %, with more than 6 cell edges = 0,411±0,014 %. Percentage of Caco2 shEpCAM#2 cells with 2 cell edges = 0,149±0,149 %, with 3 cell edges = 1,480±0,547 %, with 4 cell edges = 20,509±0,313 %, with 5 cell edges = 41,156±0,648 %, with 6 cell edges = 35,811±1,889 %, with more than 6 cell edges = 0,896±0,518 %. Unpaired *t* tests, * *P*-value <0,01. Values are mean ± s.e.m.

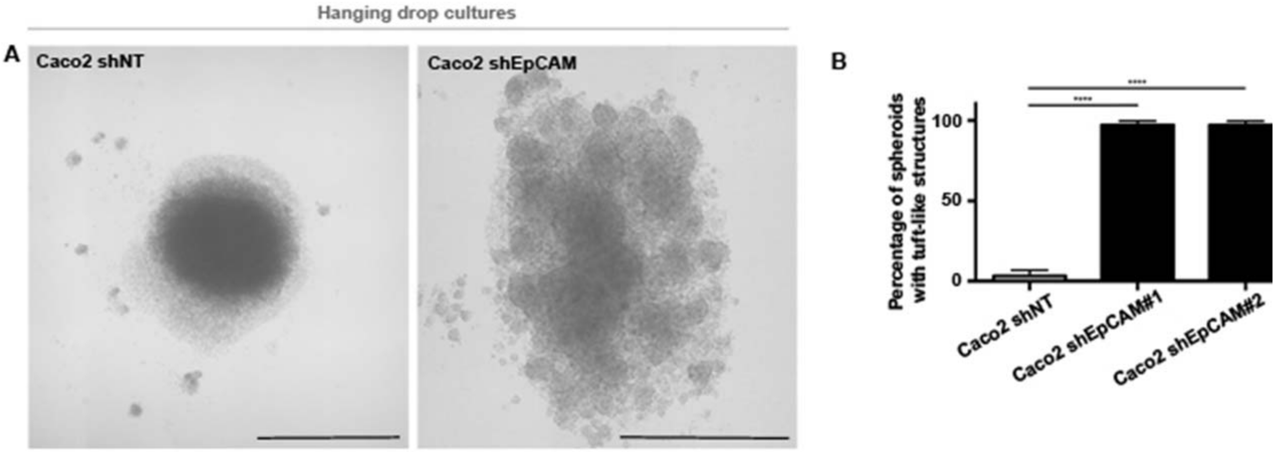

Supplementary Figure 10: Spheroid organization defects in hanging drop cultures of EpCAM-deprived cells. (A) Analyses of control (*Caco2 shNT*) or EpCAM-depleted (*Caco2 shEpCAM*) cells in hanging drop cultures for 48 hours. Scale bars, 1mm. (**B**) Statistical significance was determined using one-way ANOVA and Tukey's multiple comparison test. ****, P-value < 0.0001. Percentage of spheroids with tuft-like structures in Caco2shNT cells = 3.2 ±2.1 %, in Caco2 shEpCAM#1 cells = 97.2 ±1.5 %, and in Caco2 shEpCAM#2 = 97.1 ±1.4 %. Values are mean±s.e.m.

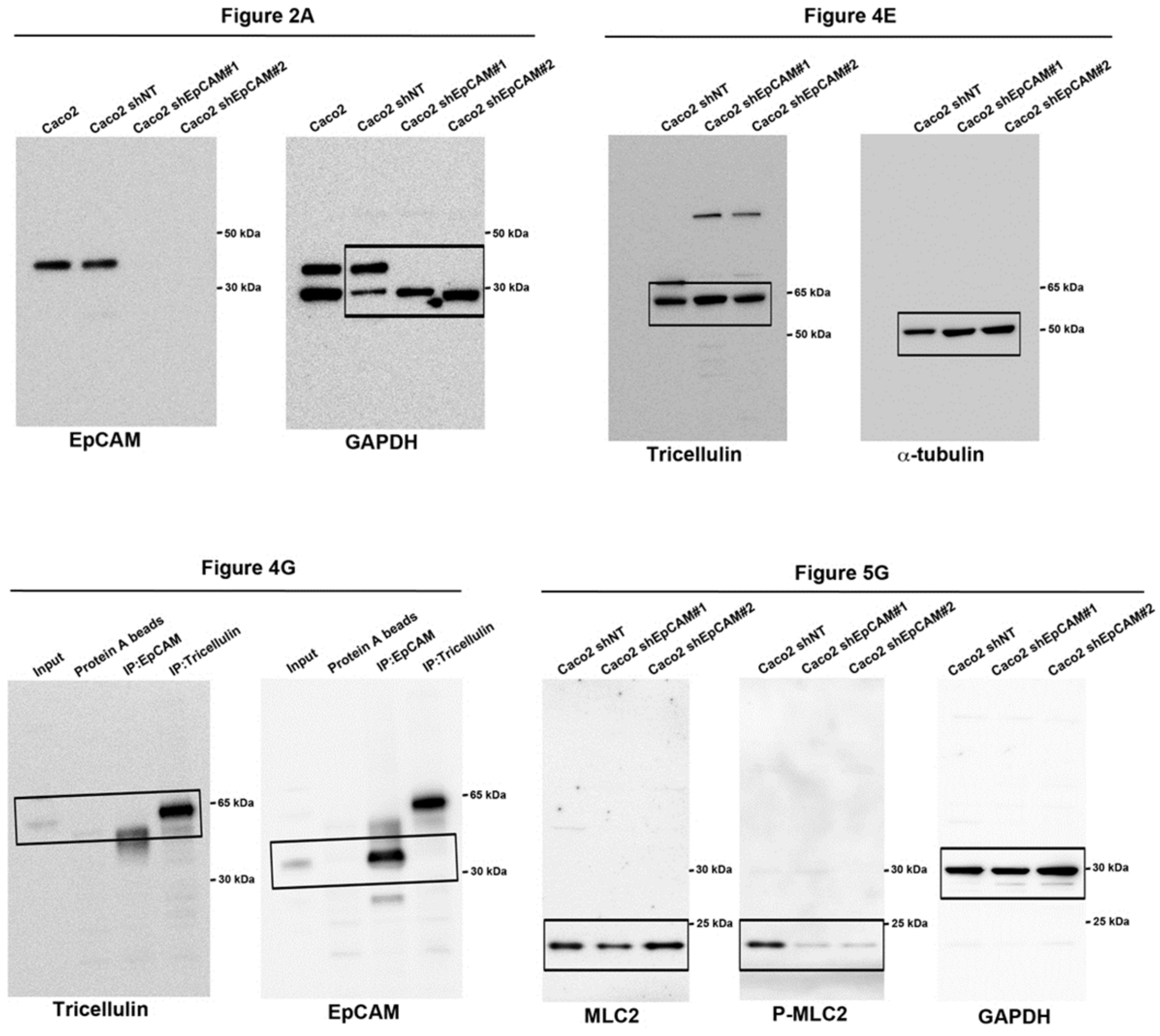

<u>Supplementary Figure 11:</u> Uncropped original gels shown in the main manuscript. Western blots correspond to those shown in Figures 2A, 4E, 4G and 5G.

Supplementary Figure 2A

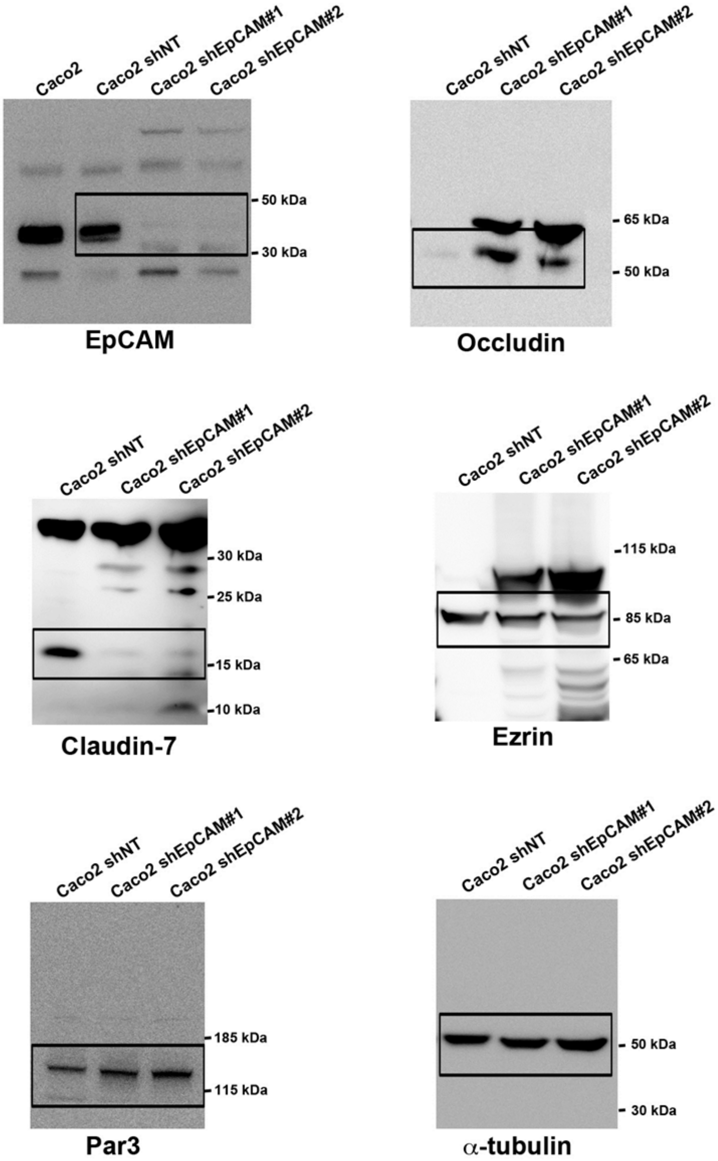

<u>Supplementary Figure 12:</u> Uncropped original gels shown in the in the Supplementary Information. Western blots correspond to those shown in Supplementary Figure 2A.

Supplementary Figure 5A

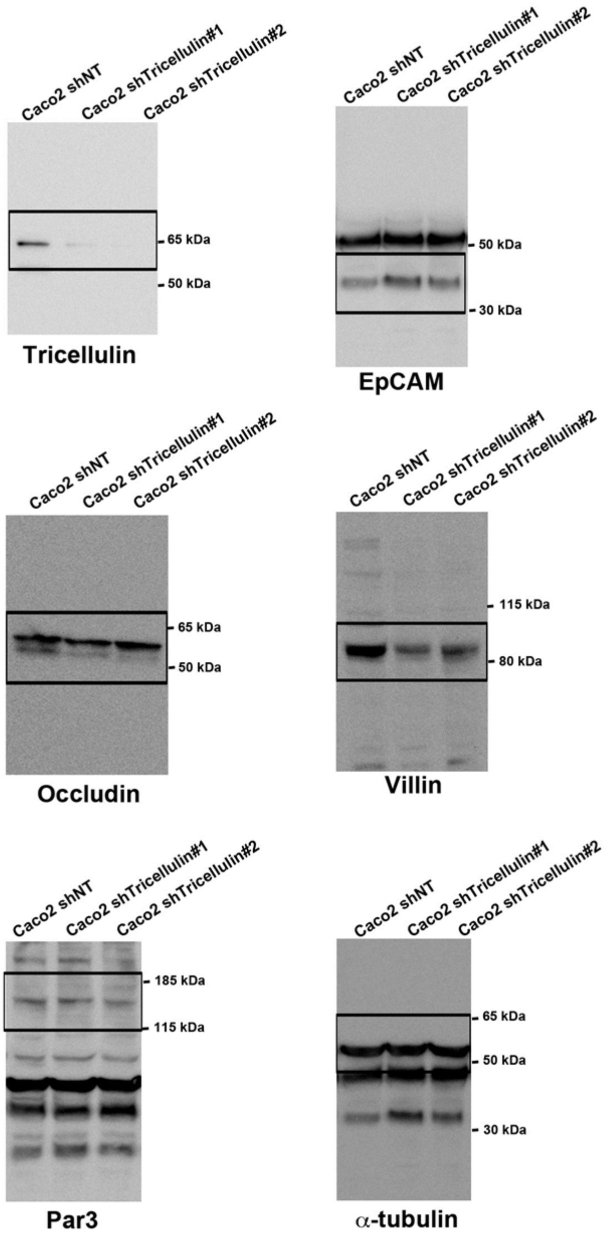

<u>Supplementary Figure 13:</u> Uncropped original gels shown in the Supplementary Information. Western blots correspond to those shown in Supplementary Figure 5A.

Supplementary Figure 7

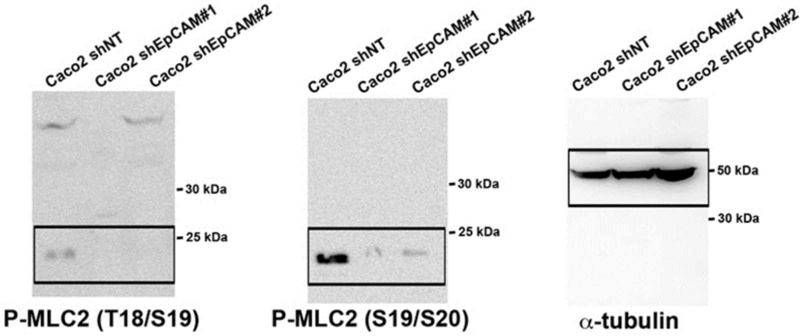

Supplementary Figure 8

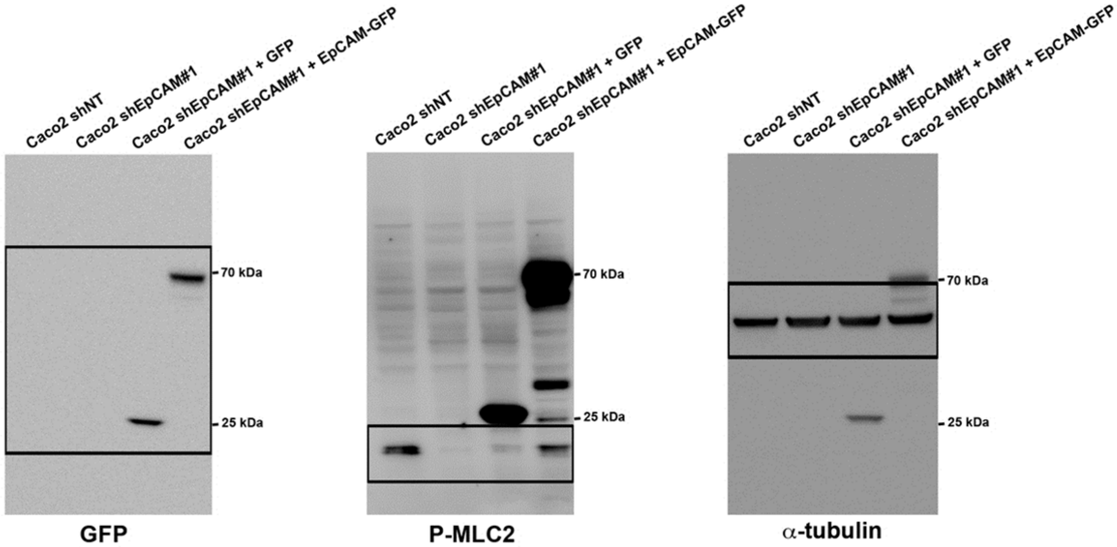

<u>Supplementary Figure 14:</u> Uncropped original gels shown in the Supplementary Information. Western blots correspond to those shown in Supplementary Figures 7 and 8.
